# Trends and Exceptions in the Interaction of Hydroxamic Acid Derivatives of Common Di- and Tripeptides with Some 3d and 4d Metal Ions in Aqueous Solution

**DOI:** 10.3390/molecules24213941

**Published:** 2019-10-31

**Authors:** András Ozsváth, Linda Bíró, Eszter Márta Nagy, Péter Buglyó, Daniele Sanna, Etelka Farkas

**Affiliations:** 1 Department of Inorganic and Analytical Chemistry, University of Debrecen, Egyetem tér 1, H-4032 Debrecen, Hungary; ozsvath.andras@science.unideb.hu (A.O.); linda.biro@science.unideb.hu (L.B.); neszma@hotmail.com (E.M.N.); buglyo@science.unideb.hu (P.B.); 2 Istituto CNR di Chimica Biomolecolare, Trav. La Crucca 3, I-07040 Sassari, Italy; Daniele.Sanna@cnr.it

**Keywords:** peptide hydroxamic acids, solution equilibrium, metal complexation, Ru(II)-, Rh(III)-based half-sandwich complexes, Mo(VI) complexes

## Abstract

By using various techniques (pH-potentiometry, UV-Visible spectrophotometry, ^1^H and ^17^O-NMR, EPR, ESI-MS), first time in the literature, solution equilibrium study has been performed on complexes of dipeptide and tripeptide hydroxamic acids—AlaAlaNHOH, AlaAlaN(Me)OH, AlaGlyGlyNHOH, and AlaGlyGlyN(Me)OH—with 4d metals: the essential Mo(VI) and two half-sandwich type cations, [(η^6^-*p*-cym)Ru(H_2_O)_3_]^2+^ as well as [(η^5^-Cp*)Rh(H_2_O)_3_]^2+^, the latter two having potential importance in cancer therapy. The tripeptide derivatives have also been studied with some biologically important 3d metals, such as Fe(III), Ni(II), Cu(II), and Zn(II), in order to compare these new results with the corresponding previously obtained ones on dipeptide hydroxamic acids. Based on the outcomes, the effects of the type of metal ions, the coordination number, the number and types of donor atoms, and their relative positions to each other on the complexation have been evaluated in the present work. We hope that these collected results might be used when a new peptide-based hydroxamic acid molecule is planned with some purpose, e.g., to develop a potential metalloenzyme inhibitor.

## 1. Introduction

Hydroxamic acids containing one or more weak acidic function(s) -C(O)N(R)OH (R = H in primary, alkyl or aryl moiety in secondary derivatives) [1,2] are an important class of organic molecules with a huge number of biological activities, including the well-known, crucial role of the hydroxamate-based natural siderophores in the microbial iron uptake [3] or antifungal, antimalarial, metalloenzyme inhibitory effects [4,5]. Some derivatives may also behave as NO donors under certain conditions [6]. In some part, these diverse and important effects also depend on the significant H-bonding ability [7] or redox behaviour of this moiety [8,9], but predominantly, the rely on its strong chelating ability [1,2]. Consequently, investigation of complexation between metal ions and hydroxamic acids is permanently the focus of interest. The most typical coordination mode of a hydroxamate function involves chelation via deprotonated hydroxyl and carbonyl oxygen atoms providing a five-membered (O,O) chelate, which can be hydroxamato, if the function is mono-deprotonated, and hydroximato, if it is doubly-deprotonated. The latter type of chelate can only be formed with primary derivatives. A few examples have also been known for other modes of interactions [8,9]. Significantly increased versatility of the potential coordination modes exists in the presence of additional donors, such as amino or/and peptide groups in a hydroxamic derivative. This is clearly demonstrated by numerous results collected mostly on metal complexes of aminohydroxamic acids [10,11,12,13,14,15,16]. Significantly, increased interest toward the metal complexes of aminohydroxamic acids have been realised following the first publication on a pentanuclear β-alaninehydroxamate-based Cu(II)-containing metallacrown complex both in solution and in the solid state [14] and the discovery of numerous and diverse possibilities of applications of such compounds [15,16]. Peptide hydroxamic acids, however, despite the special interest in them e.g., to develop substrate-based metalloenzyme inhibitors [5], have very rarely been involved in solution studies [17,18,19,20,21]. Moreover, most of the results relate to complexes with 3d^5–10^ transition metal ions and only a few with 4d metals [21]. Following our solution equilibrium work done ca. thirty years ago on the complexation of ProLeuNHOH and ProLeuGlyNHOH with several 3d metals [17], we performed a detailed equilibrium study on complexation of a few simple dipeptide hydroxamic acids with Fe(III), Al(III), Ni(II), Cu(II), and Zn(II). The special interest in ProLeuGlyNHOH is due to the fact that human collagenase (a zinc(II)-containing metalloezyme) was isolated by means of an affinity column prepared with this tripeptide hydroxamic acid [22]. The obtained results showed the exclusive formation of (O,O) chelated hydroxamate species with the hard Fe(III) and Al(III) ions, while (O,O) and (NH_2_,CO) chelated linkage isomers with Zn(II). These three metal ions were found not to induce deprotonation and parallel coordination of the peptide amide-N or primary hydroxamate-N, the processes of which, however, became deterministic with Cu(II) and especially with Ni(II) [19,20].

Out of the platinum group metals, only a recent publication from our lab discusses solution results on the rather complicated Pd(II)–AlaAlaNHOH, –AlaAlaN(Me)OH, –AlaGlyGlyNHOH, and –AlaGlyGlyN(Me)OH systems. In these systems, complexation and metal ion–induced hydrolytic and redox processes were found in a ligand-dependent extent [21]. No other metal from this group has previously been involved in investigations with peptide hydroxamic acids. The lack of interest came most probably from the high hydrolytic ability of these metals, as well as the inertness of their complexes. However, the intensive interest in relation to cancer therapy on numerous complexes with platinum group metals, together with the well-known fact that inertness of a complex is determined not only by the character of the metal ion but also by the mobility of the coordinated ligands, initiated studies in the past several years. For example, solution equilibrium measurements were successfully performed with half-sandwich type platinum group metal cations, including ruthenium and rhodium, in which an arene (benzene or its derivative such as 1-methyl-4-isopropylbenzene (*p*-cym)) or arenyl (pentamethyl-cyclopentadienyl (Cp*)) replaces three of the six solvent molecules, and complexation reactions can occur at the remaining three sites [23,24]. Recently, several types of hydroxamate-based ligands, including aminohydroxamic acids, have already been involved in solution equilibrium studies with these half-sandwich type cations [12,13], but only peptide hydroxamic acids are studied in the present work.

Likewise, according to the best of our knowledge, no publication can be found for complexes of peptide hydroxamic acids with molybdenum, despite the fact that this is an essential metal (the only one out of the 4d metals) and the biological activities of more than 50 enzymes are known to rely on it [25,26]. Again, the very high tendency of this metal for hydrolysis is one of the main reasons for the lack of investigations. Moreover, the stable form of Mo(VI) does not exist as an aqua-ion at all; only MoO_4_^2−^ ion including various protonated forms of this oxido anion or its polymeric units exist [11].

**Scheme 1:**
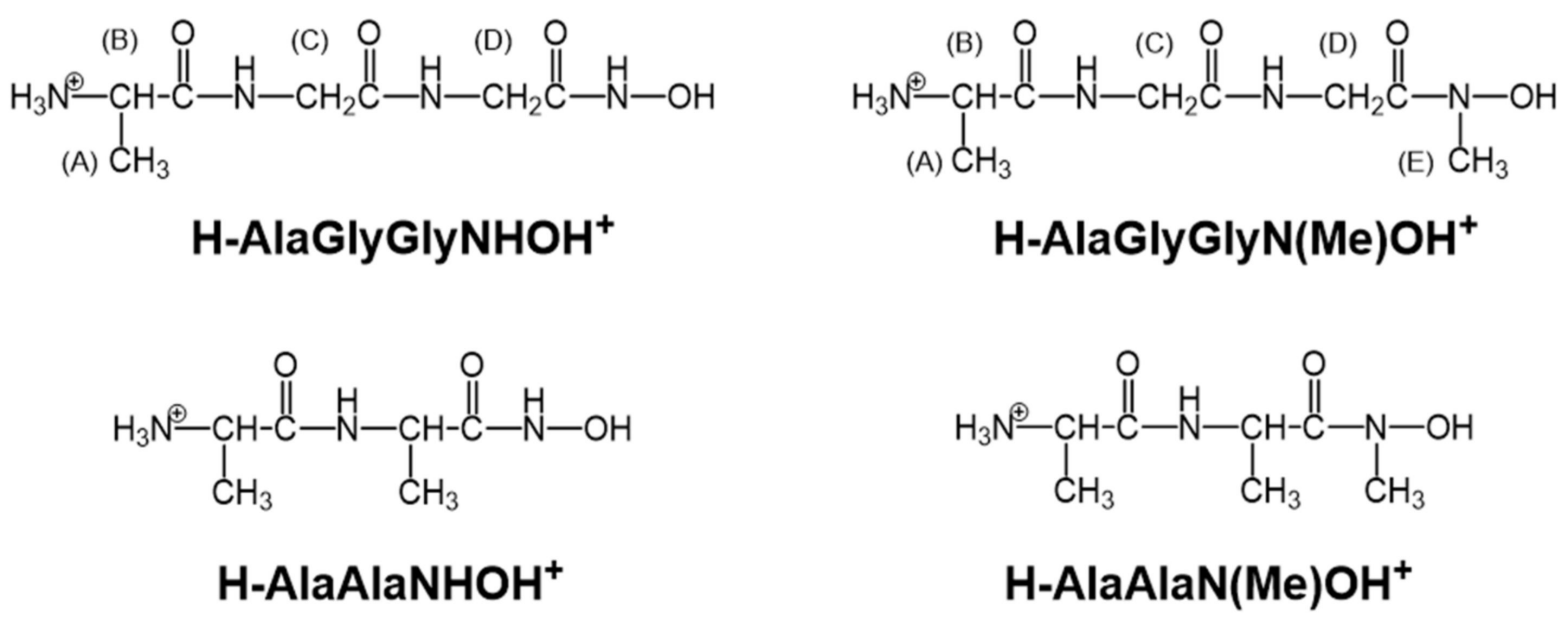
Structures of the fully protonated ligands (H_2_L^+^).

As a continuation of our previous solution equilibrium work in this subject, in the present work, we have investigated the interaction of dipeptide and tripeptide hydroxamic acids, AlaAlaNHOH, AlaAlaN(Me)OH, AlaGlyGlyNHOH, and AlaGlyGlyN(Me)OH (see their structures in Scheme 1) with 4d metals, Mo(VI), and half-sandwich type cations of ruthenium(II), as well as rhodium(III), [(η^6^-*p*-cym)Ru(H_2_O)_3_]^2+^, or [(η^5^-Cp*)Rh(H_2_O)_3_]^2+^, respectively. To provide us with the possibility for a comparison between the results collected with 3d metals and 4d ones, investigation of the complexation with the (previously not studied) above tripeptide derivatives and metal ions as Fe(III), Ni(II), Cu(II), and Zn(II) was also performed.

## 2. Results

### 2.1. Protonation Equilibria of the Investigated Peptide Hydroxamic Acids

Each of the completely protonated form of the ligands have two deprotonation processes in the measurable pH-range; the calculated dissociation constants are presented in Table 1. Literature data could be found for the investigated dipeptide derivatives only in the presence of KCl, and those are in perfect agreement with the corresponding ones in Table 1 [19,20]. As can be seen in Table 1, there is practically no difference between the corresponding values of the di- and tripeptide derivatives determined either in the presence of KCl or KNO_3_, but in accordance with earlier results, the dissociation constants for the secondary hydroxamic acids are 0.1–0.3 log unit smaller than those of the corresponding primary ones. This difference originates from the more enhanced delocalisation along the hydroxamate anions in the presence of an electron releasing alkyl group [20]. It is also known that the dissociation processes of the ammonium and hydroxamic groups considerably overlap each other [19]. Consequently, the values in Table 1 are macroconstants and cannot be ascribed unambiguously to the individual moieties. In one of our previous works, the dissociation microconstants were determined for the binding isomers of AlaAlaNHOH, and they supported the somewhat higher acidity of the ammonium group [19]. Since no significant difference can be assumed between the basicities of the investigated peptide hydroxamic acids, determination of dissociation microconstants for the additional ligands was beyond the scope of this work.

### 2.2. Complexation of AlaGlyGlyNHOH and AlaGlyGlyN(Me)OH with Selected 3d Ions—Fe(III), Ni(II), Cu(II), and Zn(II)

Although the present solution equilibrium work has focused on the complexation of hydroxamic acid derivatives of common di- and tripeptides with the biologically important 4d metals Mo(VI), Ru(II), and Rh(III), a comparison between the results collected on complexes with the selected 3d and 4d metals has also been our aim.

Since detailed solution equilibrium results have been previously published only on the Fe(III), Ni(II), Cu(II), and Zn(II) complexes of the two dipeptide hydroxamic acids AlaAlaNHOH and AlaAlaN(Me)OH [19,20], the present paper shortly discusses the results obtained for the two tripeptide hydroxamic acids AlaGlyGlyNHOH and AlaGlyGlyN(Me)OH. 

The experimental results were collected on the Fe(III), Ni(II), Cu(II), and Zn(II) complexes of AlaGlyGlyNHOH and AlaGlyGlyN(Me)OH by the methods and under the conditions detailed in the Experimental section. The equilibrium models and logarithmic overall stability constants presented in Table 2 were determined by pH-potentiometry, except for the complexes with Fe(III), where combination of pH-metry and spectrophotometry provided the possibility for calculation of stability constants. The reason behind is the formation of complexes with [FeHL]^3+^ stoichiometry below the pH-metrically measurable pH-range (far below pH 2). Their stability constants therefore were determined by fitting the spectra recorded on individual samples in the pH-range 0.7–1.6 (see Experimental part). These calculated constants were kept fixed when pH-metric titration curves, and UV-Visible spectra registered in the whole measured pH-range were fitted. 

For easier comparison, Table 2 also contains the previously published data for the complexes of dipeptide derivatives determined under the same conditions as used in the present study. 

As shown in Table 2, there is a good agreement between both the equilibrium models and numerical values of the constants determined by pH-metry and spectrophotometry for the mono-, bis- and tris-complexes of primary and secondary di- and tripeptide derivatives with Fe(III). The only difference between the models is that the [FeL]^2+^ complex—what was found with the dipeptide hydroxamic acids and was assumed to be a mixed hydroxido species—was not formed in measurable concentration with the two tripeptide derivatives. Based on the almost perfect agreement, the same coordination modes are assumed with the tripeptide derivatives as it was found with the dipeptide-based counterparts [19,20]: coordination of the hydroxamate oxygens while the non-coordinating amino moiety is protonated. The UV-Visible spectra showing the well-known charge-transfer (C.T.) bands in both Fe(III)-tripeptide hydroxamic acid systems, clearly support this assumption. The registered spectra in the pH-range ca. 0.7–1.6 (where the [FeHL]^3+^ is formed) are characteristic for an Fe(III)-monohydroxamato complex (λ_max_ = 510 nm). Upon increasing the pH, a decrease of the λ_max_ down to 460 nm in a second stepwise process by pH ca. 3–3.5 occurs (this process belongs to the formation of the bis-hydroxamato species [Fe(HL)_2_]^3+^), while as a result of a third process, the tris-hydroxamato species [Fe(HL)_3_]^3+^ is formed by ca. pH 6.5 with a characteristic λ_max_ = 420–425 nm. Above pH ca. 6.5, the formation of mixed hydroxido species was indicated to start by the spectra even at 1:5 metal to ligand ratio in both systems. 

Zn(II) is the other metal ion, with which both the equilibrium models and stability constants are quite similar in all of the four systems. The only exception in the models is that bis-complex was found to form under our conditions with the dipeptide derivatives but not with the two tripeptide hydroxamic acids, most probably because of sterical reasons. All these findings suggest that Zn(II) behaves very similarly toward the two tripeptide-based ligands, as was previously supported by ^1^H-NMR results for the complexes of AlaAlaNHOH [19] and AlaAlaN(Me)OH [20]. Consequently, AlaGlyGlyNHOH and AlaGlyGlyN(Me)OH are assumed to form moderate stability binding isomers with Zn(II), in which coordination occurs via either the five-membered (O,O)_hydr_ chelate or the five-membered (NH_2_,CO) chelate. Only one of these two possible chelates exists in [ZnHL]^2+^ (which starts to form at pH ca. 4.8–5), while the other moiety is still in protonated, non-coordinated form. Upon increasing the pH, the formation of [ZnL]^+^, followed by [ZnH_−1_L], was found. They are most probably mixed hydroxido species and in the pH-range where the free ligand itself deprotonates (above pH 7) and the dissociation of the non-coordinating moiety also occurs. As in the case of Fe(III), there is no indication for the Zn(II)-induced deprotonation and coordination of peptide amide function in any of the investigated systems. 

**Figure 1:**
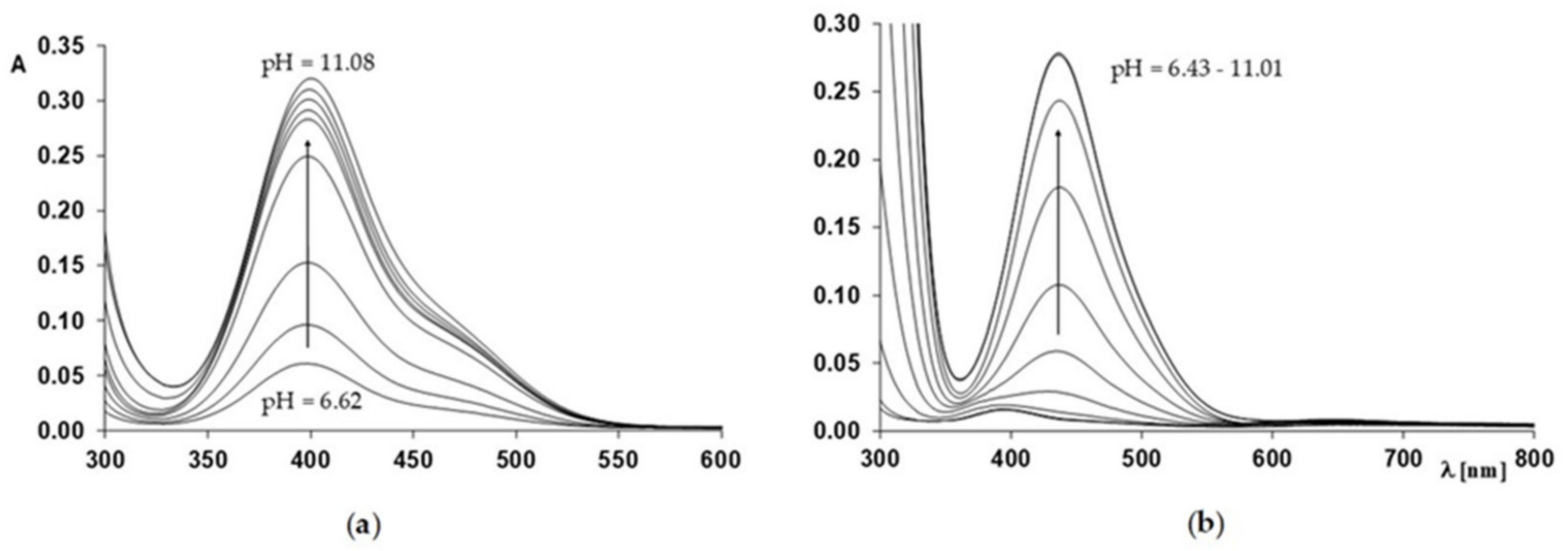
UV-Visible spectra recorded at different pH values for the (**a**) Ni(II)–AlaGlyGlyNHOH and (**b**) Ni(II)–AlaGlyGlyN(Me)OH systems at 1:1 ratio (c_Ni(II)_ = 1.5 mM).

The situation is completely different with Ni(II) and Cu(II), in which metal ions accept N-donor ligands readily. At the same time, the different number and, in some part, type of the N-donors in the studied ligands results in different coordinating behaviour of them toward these metal ions: AlaAlaNHOH contains 3N (NH_2_, N_amide_, N_hydr._), AlaAlaN(Me)OH involves 2N (NH_2_, N_amide_) donor atoms, there are 4N donors in the tripeptide derivative AlaGlyGlyNHOH (NH_2_, 2×N_amide_, N_hydr._), while 3N in its secondary counterpart, AlaGlyGlyN(Me)OH (NH_2_, 2×N_amide_). The significant role of the amino-N, amide-N and for the primary hydroxamic derivatives, hydroxamate-N in the Ni(II)– and Cu(II)–AlaAlaNHOH and –AlaAlaN(Me)OH systems was discussed in our previous papers [19,20]. In the present work, both AlaGlyGlyNHOH and AlaGlyGlyN(Me)OH were found to react with Ni(II) above pH ca. 4. The first species, [NiHL], are formed in low extent below pH ca. 6, in which the ligands are assumed to coordinate in a similar manner as it was supported with the dipeptide derivative, either via five-membered (NH_2_, CO) or (O,O)_hydr_. The colour of the samples turned to yellow above pH ca. 6 with the primary derivative and above pH ca. 7 with its secondary counterpart. This latter pH is much lower than the value, where the color turned to yellow in the Ni(II)–AlaAlaN(Me)OH system, above pH 9 [20]. Above these pH values, the spectra registered in both systems are characteristic for square planar Ni(II) complexes (Figure 1a,b). 

Although the most probable cooperative processes with the primary ligand were too slow to allow determination of stability constants for the stepwise equilibria, there is no doubt that four equivalents of base are consumed by the end of the titrations, up to pH ca. 11. The calculated stability constant for [NiH_−2_L]^−^ formed is shown in Table 2. The two dissociable protons of the ligand consume two equivalents of base, while the additional two react with the protons from the two peptide moieties. Consequently, [NiH_−2_L]^−^, which was found to be a mixed hydroxido complex with AlaAlaNHOH [19], is a 4N-coordinated complex with AlaGlyGlyNHOH, and the coordinating donor set is (NH_2_,N_amide_,N_amide_,N_hydr_). This assumption is further supported by the UV-Visible parameters (λ_max_ = 400 nm, ε ca. 200 M^−1^cm^−1^), which are similar to those obtained previously for the 4N-coordinated complex in the Ni(II)–ProLeuGlyNHOH system [17]. 

Like in the case of the primary counterpart, four equivalents of base were altogether consumed during the titration of Ni(II)–AlaGlyGlyN(Me)OH samples as well. There is no doubt that the final species, [NiH_−2_L]^−^, is a planar complex in this case, in which the metal ion is coordinated by the (NH_2_, N_amide_,N_amide_,O_hydr_) donor set. With this secondary tripeptide hydroxamic acid, however, the processes were fast enough to allow the calculation of stability constants for the intermediate species. At the same time, if the stepwise dissociation constants for the complexes using the corresponding overall stability constants in Table 2 are calculated, an irregular trend is found: pK_NiL_ is higher than pK_NiH−1L,_ (5.04 − (−3.3) = 8.34 and −3.3 − (−10.78) = 7.48, respectively), showing cooperativity of the processes in this system, too. With AlaGlyGlyN(Me)OH, no hydrolysis, but bis-complex formation in a small extent and in a narrow pH-range (what was dominant with the secondary dipeptide, AlaAlaN(Me)OH [20]), was detected. 

Between Cu(II) and the two tripeptide derivatives, complexation starts as low as pH ca. 2.5–3, where the competition between the proton and metal ion for the coordinating sites is very significant, as it was discussed previously [27]. An evaluation, based on this literature information allowed us to draw the conclusion that among the likely chelates (O,O)_hydr_ has the highest conditional stability under the above acidic conditions. Consequently, the hydroxamate function behaves as anchor site for the Cu(II) ion. In fact, this coordination mode was unambiguously supported by both the characteristic charge transfer band at λ_max_ ca. 370–380 nm and the EPR results (g_II_ = 2.345 and 2.335, A_II_ = 160.5 × 10^−4^ and 159.8 × 10^−4^ cm^−1^ with AlaGlyGlyNHOH and AlaGlyGlyN(Me)OH, respectively) in the [CuHL]^2+^ formed. Despite the fact that four equivalents of base were consumed with both of the ligands by pH ca. 11, several experimental findings collected above pH ca. 4.5 in the two systems differ from each other. Namely: (1) The primary derivative was capable of binding Cu(II) excess, the secondary not. (2) Bis-complexes were less favoured with the primary ligand but were present in low concentration also with this ligand. (3) The processes were very slow above pH 5 in the Cu(II)–AlaGlyGlyNHOH system, hindering the calculation of stability constants for some of the species formed, but those were significantly faster for the Cu(II)–AlaGlyGlyN(Me)OH system allowing calculations from the whole measured pH-range (see Table 2). Fortunately, EPR and UV-Visible measurements provided clear support for the different complexes formed in these two systems. For illustration, some EPR spectra recorded at various pH values for Cu(II)–AlaGlyGlyNHOH and Cu(II)-AlaGlyGlyN(Me)OH samples are shown in Figure S1 (a) and (b), respectively.

As Figure S1a reveals, with AlaGlyGlyNHOH in the pH-range ca. 6–7, the decreased intensity of the EPR signals supports the presence of a dinuclear species as major complex following [CuHL]^2+^ with hydroxamate coordination. The existence of some EPR activity indicates that no spin-crossover but weak interaction exists between the two metal ions in the dinuclear complex. The calculated EPR parameters suggest that one of the Cu(II) is coordinated by two (O,O)_hydr_ chelates (g_II_ = 2.283 and A_II_ = 181.7 × 10^–4^ cm^−1^), while the other is coordinated by nitrogens. At pH = 9.2, the calculated parameters (g_II_ = 2.176 and A_II_ = 213.4 × 10^–4^ cm^−1^) are in perfect agreement with those published for the 4N-coordinated complex in the Cu(II)–ProLeuGlyNHOH system (g_II_ = 2.181 and A_II_ = 208.5 × 10^−4^ cm^−1^) [17]. This suggests an (NH_2_,N_amide_,N_amide_,N_hydr_)-type coordination mode in [CuH_–2_L]^–^, predominating at this pH. Additional support for the suggested coordination mode in this complex was provided by the λ_max_ = 500 nm of the *d-d* band in the UV-Visible spectrum. 

**Scheme 2:**
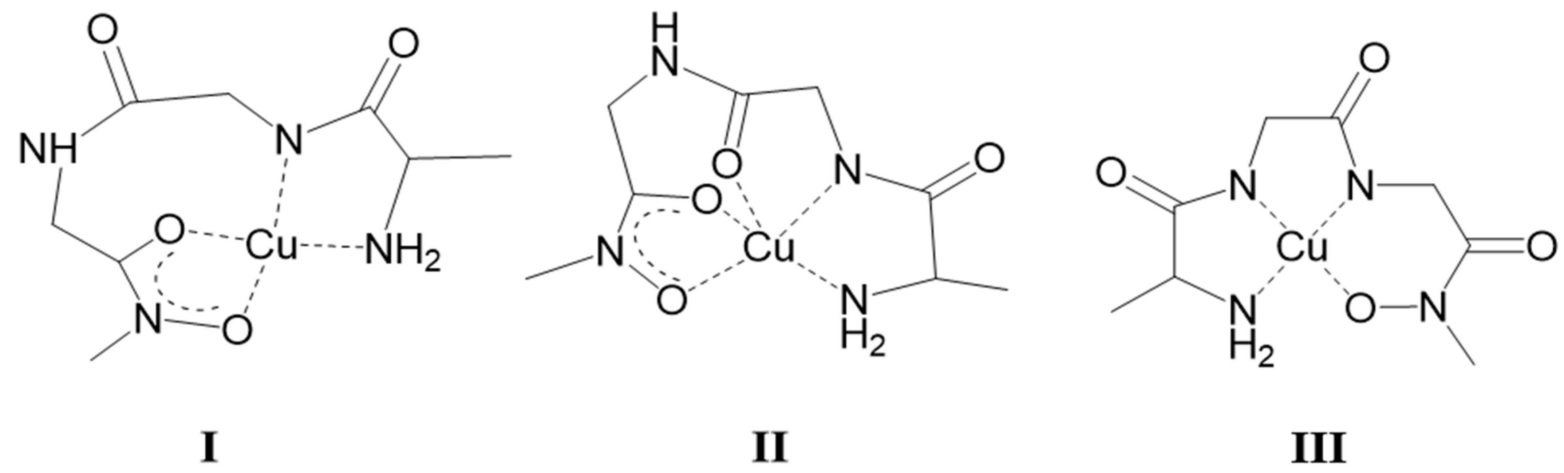
Plausible binding modes in the complexes [CuH_–1_L] (structures I and II) and the most possible mode in [CuH_−2_L]^–^ (structure III), L^–^ = AlaGlyGlyN(Me)O^–^.

Interestingly, the mononuclear bis-complex, [Cu(HL)_2_]^2+^ was found as a major species at ligand excess in the pH-range 4.7–6.5 with AlaGlyGlyN(Me)OH (see Figure S1b). The EPR parameters calculated for this complex show coordination of two hydroxamates (g_II_ = 2.283 and A_II_ = 181.7 × 10^–4^ cm^–1^). In the pH-range 6.5–8.1 involvement of nitrogens in the coordination is clearly shown by the EPR spectra, but the presence of the C.T. band at λ_max_ = 380 nm in this pH range proves the involvement of the hydroxamate oxygens in the coordination too. Some cooperativity of the processes in this pH-range is indicated by the results and in [CuH_-1_L], predominating at 1:1 metal ion to ligand ratio, highly distorted arrangement of the coordinated donors is shown by the appropriate EPR parameters (g_II_ = 2.172 and A_II_ = 124.3 × 10^−4^ cm^−1^). Tentative binding modes for this species are shown in structures I and II in Scheme 2. The combination of g and A values suggests that the donor atoms involved in the metal coordination are strong donors (low g value) but the structure is very distorted (low A value); if also the g value was high we should think to a weak coordination [28,29]. Because we do not have a comparison with similar complexes having the same donor atoms coordinated but with a less distorted structure, we are not sure that the coordination is exactly the one that is reported as proposed structures I or II in Scheme 2. Upon increasing the pH further, [CuH_−2_L]^−^ becomes even more predominant with the (NH_2_,N_amide_,N_amide_,O_hydr_) donor set (structure III in Scheme 2). 

### 2.3. Complexation of AlaAlaNHOH, AlaAlaN(Me)OH, AlaGlyGlyNHOH, and AlaGlyGlyN(Me)OH with the Essential 4d Ion Mo(VI)

**Figure 2:**
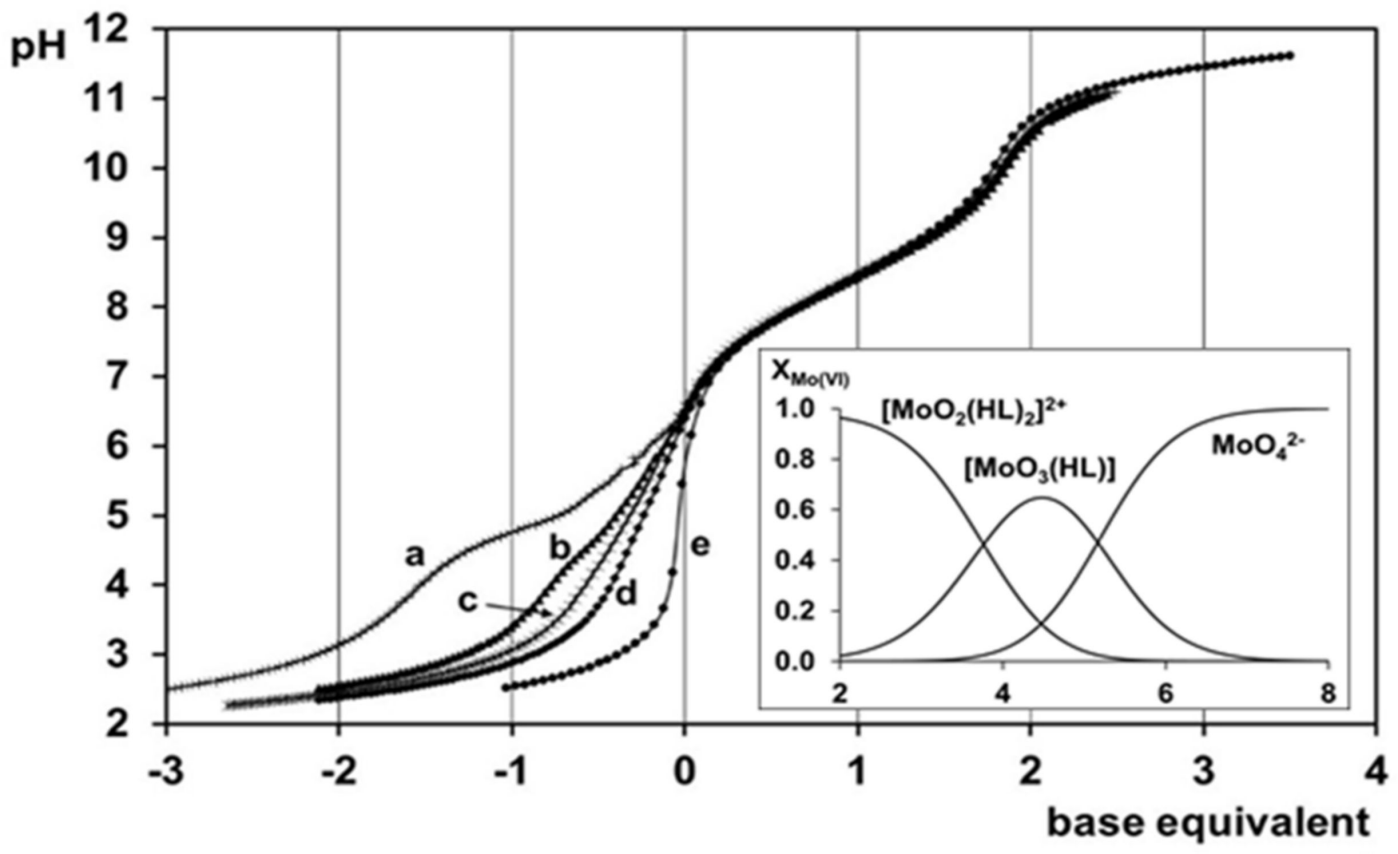
Titration curves of the Mo(VI)–AlaAlaNHOH system at (**a**) 1:1, (**b**) 1:2, (**c**) 1:3 and (**d**) 1:4 metal ion to ligand ratio with the titration curve of the (**e**) H^+^–AlaAlaNHOH system (c_L_ = 6.00 mM) and in inset the calculated concentration distribution curves for the Mo(VI)–AlaAlaNHOH system at 1:4 metal ion to ligand ratio (c_L_ = 1.82 mM; negative base equivalents refer to an excess of acid in the samples).

The registered pH-potentiometric titration curves, similarly to the few previous results on Mo(VI)-monohydroxamic acid systems [30], indicated very strong interaction between the studied ligands and Mo(VI), but only under acidic conditions. No difference between the curves for the ligands or those registered in the Mo(VI)-containing samples was observed above pH ca. 7.5. To illustrate this, Figure 2 shows the titration curves registered for AlaAlaNHOH alone and for the Mo(VI)–AlaAlaNHOH system at various metal ion to ligand ratios.

All the titration curves can be fitted well (see fitting parameters in Table 3) for all the studied systems with the model involving the proton complexes of the ligands, hydroxido species of molybdenum(VI) (see the Experimental section), as well as the metal complexes [MoO_2_(HL)_2_]^2+^ and [MoO_3_(HL)]. The calculated overall stability constants of the species formed in equilibrium (1) are presented in Table 3. As an example, the inset in Figure 2 shows the concentration distribution curves of complexes formed in the Mo(VI)–AlaAlaNHOH system based on pH-metric results (similar curves were obtained for the Mo(VI)–AlaAlaN(Me)OH system).

MoO_4_^2^^−^ + xL^−^ + 3xH^+^ ⇌ [MoO_4−x_(HL)_x_]^(x−1)+^ + xH_2_O (1)

where x = 1 or 2.

Table 3 shows no significant differences between the stability constants determined in the four systems for the two types of complexes, [MoO_2_(HL)_2_]^2+^ or [MoO_3_(HL)], respectively. However, our previous results on the few Mo(VI)–monohydroxamic acid systems revealed that there were some processes that were not accompanied by pH-effect at all [30]. Consequently, pH-potentiometry alone could not detect all the solution equilibrium processes. Fortunately, additional information could be obtained using UV-Visible spectrophotometry, ^1^H-NMR, and ^17^O-NMR methods. Using these methods, the following results were obtained. 

**Figure 3:**
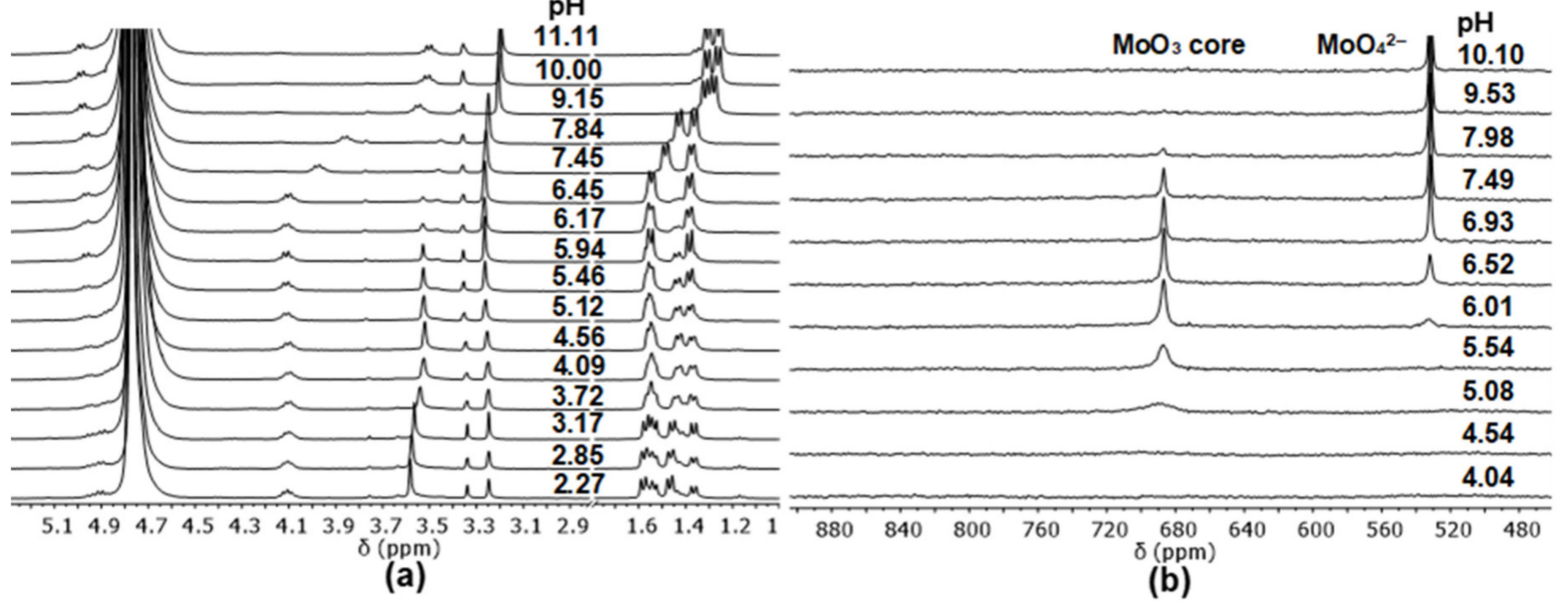
pH dependence of (**a**) ^1^H-NMR (c_L_ = 0.015 M) and (**b**) ^17^O-NMR (c_L_ = 0.15 M) spectra of the Mo(VI)–AlaAlaN(Me)OH system at 1:3 metal ion to ligand ratio. (^1^H-NMR signal at 3.34 ppm refers to a few percent methanol impurity in the ligand).

**Figure 4:**
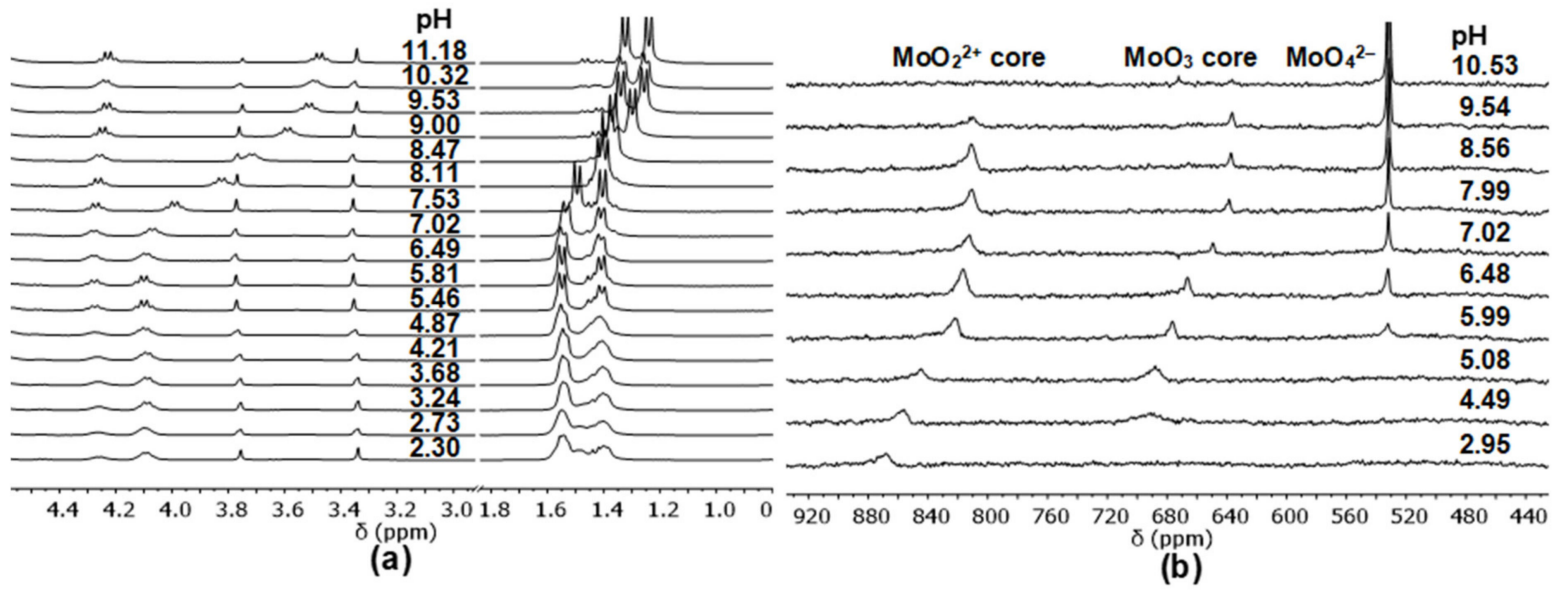
pH dependence of (**a**) ^1^H-NMR (c_L_ = 0.015 M) and (**b**) ^17^O-NMR (c_L_ = 0.15 M) spectra of the Mo(VI)–AlaAlaNHOH system at 1:3 metal ion to ligand ratio. (^1^H-NMR signal at 3.34 ppm refers to few percent methanol impurity in the ligand).

A high intensity C.T. band at λ_max_ ca. 290 nm could be detected in all the studied systems as low as pH 2, which started to decrease above pH 5. This band is known to refer to a molybdenum-dioxido-bis-hydroxamato species [30], so this finding, together with those based on ^1^H-NMR measurements, clearly supports the predominance of hydroxamate coordinated species with all the four di- and tripeptide hydroxamic acids having the amino-moiety in non-coordinated, protonated form. Beside this very important similarity in the behaviour of the four ligands toward Mo(VI), the ^1^H-NMR and especially ^17^O-NMR spectra also indicated significant differences between the interactions with the primary and secondary derivatives. This is well demonstrated by the comparison of Figure 3a,b where ^1^H-NMR spectra are presented, and Figure 4a,b where ^17^O-NMR spectra recorded at various pH for the Mo(VI)–AlaAlaN(Me)OH and Mo(VI)–AlaAlaNHOH systems, respectively. (Similar experimental results were obtained for the corresponding tripeptide-based counterparts; those are not shown).

As Figure 3a shows, complexation processes between Mo(VI) and the secondary derivative are slow enough on the NMR time scale to obtain individual signals for the non-dissociable protons locating close to the coordinating sites in the complexes compared to those for the free ligand. The fractions calculated from the relative intensities of these signals at pH 2 (ratio of the complexed and non-complexed ligand is 2:1) are in perfect agreement with the assumption that at this pH [MoO_2_(HL)_2_]^2+^ exists exclusively in measurable concentration, while one third of the ligand remains in non-complexed form. Some broadening of the signals belonging to the complex is observable in the spectra above pH 4 and, parallel, small but measurable decrease in their chemical shift, either. This is the pH, where [MoO_3_(HL)] is present in measurable concentration by the pH-metrically determined concentration distribution curves (see inset in Figure 2) and where the well-defined characteristic signal of the MoO_3_-core at 680 ppm in the ^17^O-NMR spectrum can already be detected in Figure 3. Various exchange processes might cause the disappearance of the characteristic signal of the MoO_2_^2+^ core [30]. The intensity of the ^1^H and ^17^O signals belonging to the MoO_3_-containing species starts decreasing at pH ca. 6 and 7, respectively, and they disappear by pH ca. 7.5–8. Above this pH, only the existence of MoO_4_^2−^ ions and the free secondary ligand are supported by all the used methods. Consequently, these secondary peptide-based hydroxamic acids interact with Mo(VI) in the acidic and neutral pH-range, but they are not in the coordination sphere above neutral pH, where only MoO_4_^2−^ and the free ligand exist.

With primary derivatives, the situation is different. Although pH-potentiometry showed similar complexation behaviour with all the four studied ligands, ^1^H and ^17^O-NMR results indicate significant differences (*cf*. Figure 3 and Figure 4). Very broad ^1^H-NMR signals can be seen in Figure 4a for the Mo(VI)–AlaAlaNHOH system in the acidic region and sharpening of them at higher pH. Similar observation was made in the Mo(VI)–acetohydroxamic acid system, where, as a function of pH, various inter- and intramolecular exchange processes were suggested—faster ones in the acidic region, and formation of more inert species at higher pH [30].

^17^O-NMR spectra of the Mo(VI)–AlaAlaNHOH system were recorded between pH ca. 3.0–10.5 at a metal ion to ligand ratio 1:3. The spectra in Figure 4b provide the following information.

1. Although the characteristic signal of MoO_4_^2–^ appears at pH ca. 6 with this primary derivative (the same is seen in Figure 3b with the secondary counterpart), to some extent, the primary ligand remains in coordinative interaction with the MoO_2_^2+^-core up to pH ca. 10. Moreover, the pH-dependence of the chemical shift of the quite broad signals of the complexes with MoO_2_^2+^-core shows two inflexion points (two deprotonation processes). These experimental results support the formation of three different species with this core as a function of pH.
2. The very broad NMR signals of MoO_3_ oxygens are clearly observable at ca. 688 ppm at pH 4.49, and their chemical shifts show one inflexion point as a function of pH (indicating one deprotonation step and two types of species with MoO_3_ core). Furthermore, its intensity starts decreasing above pH 7 and almost disappears by pH 10.5.

Fitting the chemical shifts vs. pH curves by the computer program PSEQUAD provided the following dissociation constants (p*K*): p*K*_1_ = 4.1(4), p*K*_2_ = 5.7(1) for the MoO_2_^2+^-containing species and p*K* = 6.52(3) for the MoO_3_-containing one. The corresponding values with the previously studied primary acetohydroxamic acid were p*K*_1_ = 4.45, p*K*_2_ = 6.74, and p*K* = 7.73, respectively [30]. There is no doubt that similar processes can be suggested with the primary peptide hydroxamic acids as were found with acetohydroxamic acid [30], in which two factors (strong hydrogen bonding character of a hydroxamate-NH and possibility of deprotonation of it resulting in the formation of a hydroximate chelate with very high stability) play a crucial role. The suggested two processes are shown in Equations (2) and (3) with MoO_2_^2+^ core and in Equation (4) with MoO_3_.

[MoO_2_(HL)_2_]^2+^ + 2H_2_O ⇌ [MoO_2_(HL)(OH)_2_] + H_2_L^+^ + H^+^ (2)

[MoO_2_(HL)(OH)_2_] ⇌ [MoO_2_(HLH_–1_)(OH)_2_]^−^ + H^+^ (3)

[MoO_3_(HL)(H_2_O)] ⇌ [MoO_3_(HLH_–1_)(H_2_O)]^−^ + H^+^ (4)

where HL refers to the ligand coordinated via the hydroxamate chelate and the amino moiety is still protonated. HLH_−1_^−^ symbolizes doubly deprotonated hydroximate function, with protonated amino end.

**Scheme 3:**
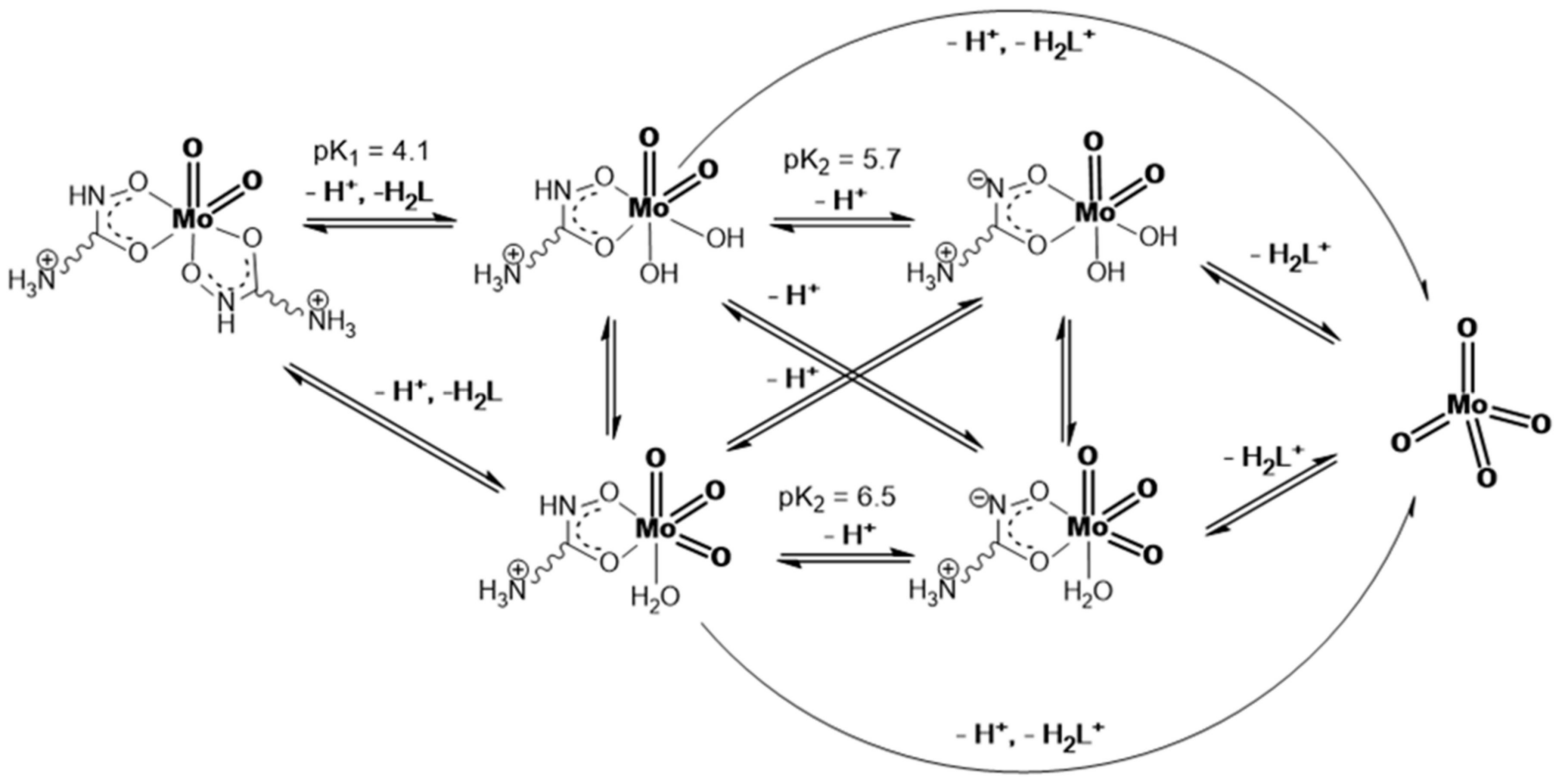
The most likely equilibrium processes as a function of pH in Mo(VI)—primary peptide hydroxamic acid systems.

For the investigated Mo(VI)-primary peptide hydroxamic acid systems, the various most likely equilibrium processes are summarized in Scheme 3. Remarkable intramolecular exchange processes between the different species shown above (including water) result in significant broadening of the signals, as was detailed in our previous paper [30].

### 2.4. Complexation of AlaAlaNHOH or AlaAlaN(Me)OH with Two Half-Sandwich Type Cations—[(η^6^-p-cym)Ru(H_2_O)_3_]^2+^ or [(η^5^-Cp*)Rh(H_2_O)_3_]^2+^

**Figure 5:**
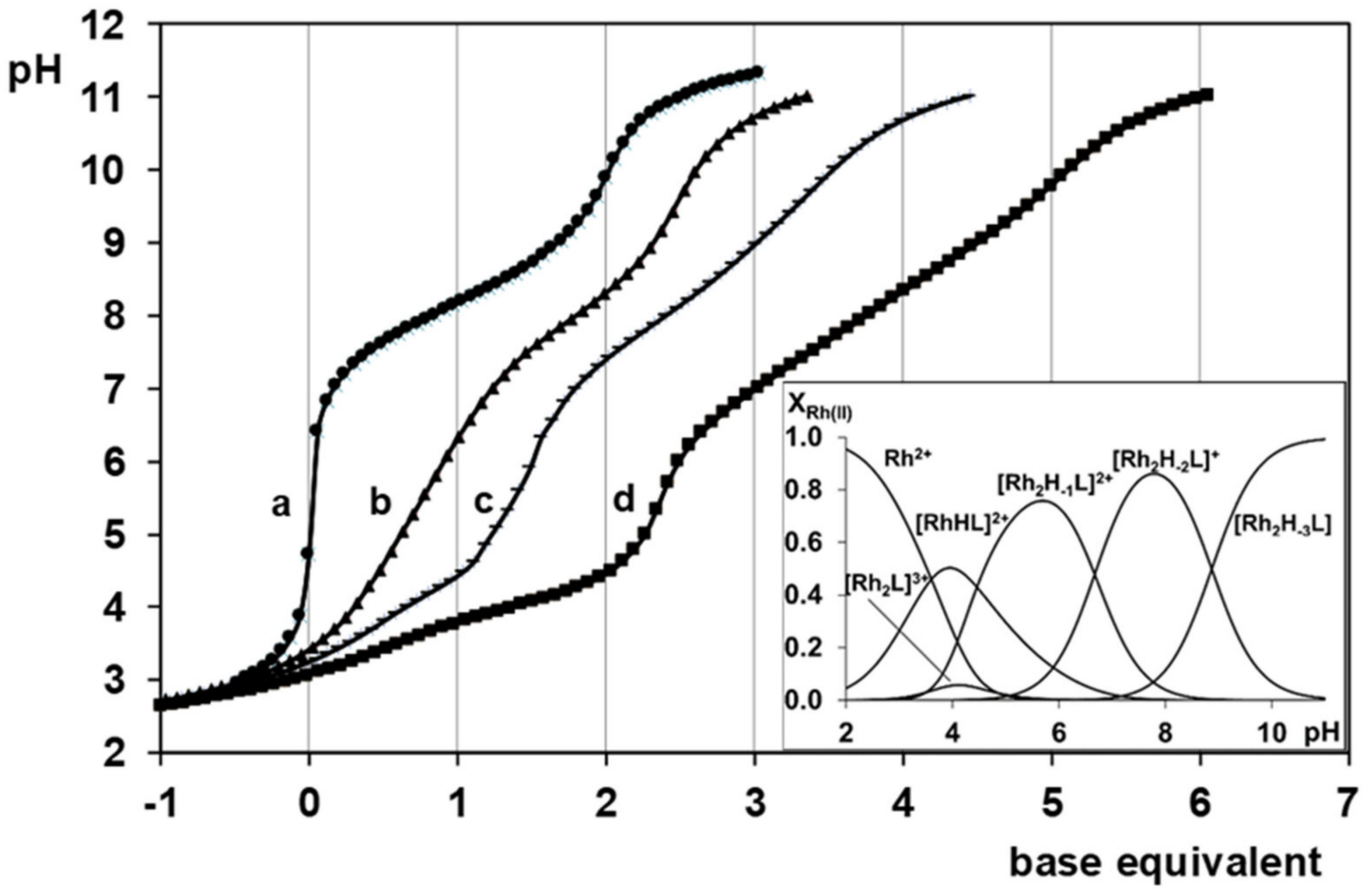
Titration curves of (**a**) the H^+^‒AlaAlaNHOH system and the [(η^5^-Cp*)Rh(H_2_O)_3_]^2+^‒AlaAlaNHOH systems at (**b**) 1:2, (**c**) 1:1 and (**d**) 2:1 ratios (c_L_ = 1.62 mM) with the related concentration distribution curves in inset calculated at 1:1 metal ion to ligand ratio (c_L_ = 2.00 mM; negative base equivalent refers an excess of acid in the sample; “Rh” stands for [(η^5^-Cp*)Rh(H_2_O)_3_]^2+^).

The speciation profiles of the complexes formed in these systems were determined via a combined use of pH-potentiometry, ^1^H-NMR, and ESI-MS. Fortunately the complex formation processes were fast enough in all of the systems to apply conventional pH-potentiometry; for details see the Materials and Methods section. Because the titration curves registered for the two systems containing the primary derivative, AlaAlaNHOH, were similar to each other. As a representative example, the curves registered for the [(η^5^-Cp*)Rh(H_2_O)_3_]^2+^-containing samples and the free ligand within the pH-range 2–11, together with the concentration distribution curves in inset, are depicted in Figure 5.

The best fit of the titration curves was obtained with the model and stability constants collected in Table 4.

**Scheme 4:**
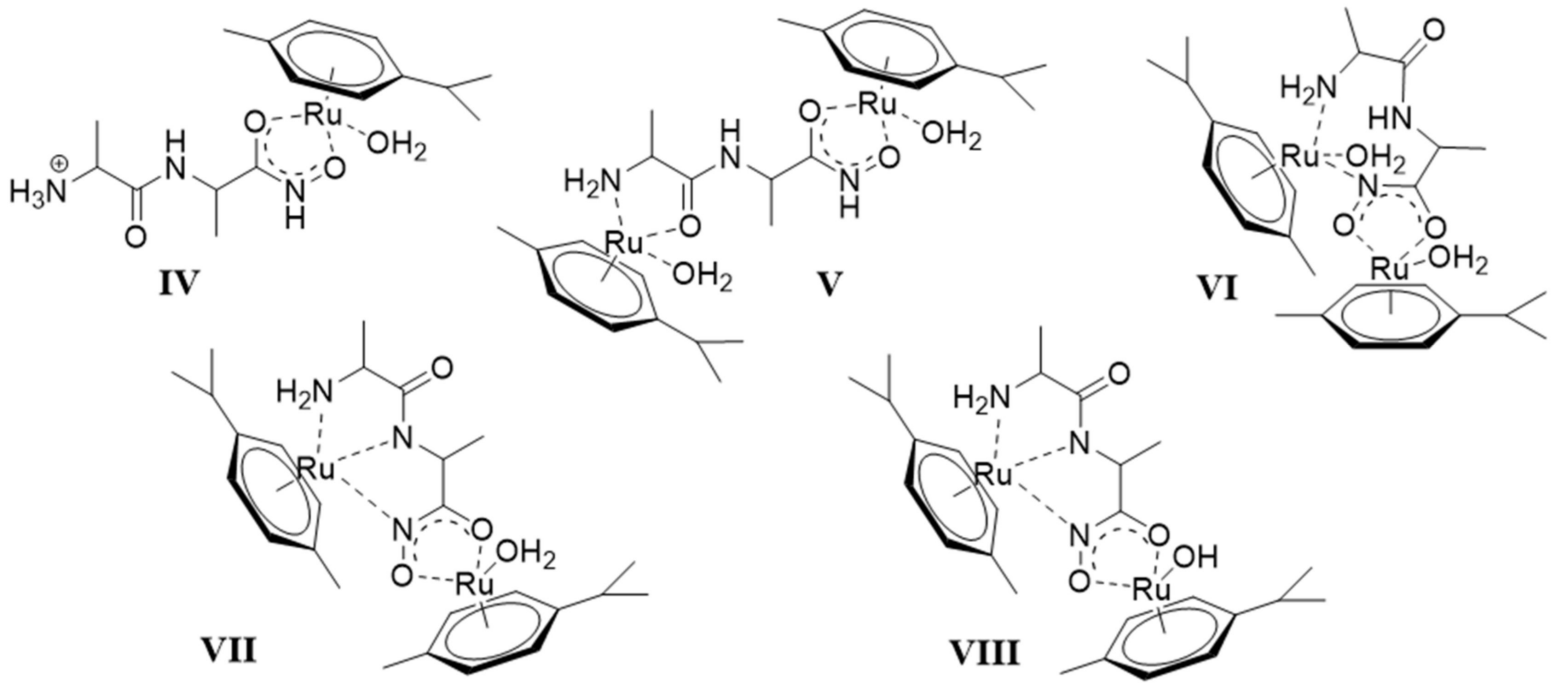
Suggested structures for the complexes formed in [(η^6^-*p*-cym)Ru(H_2_O)_3_]^2+^–AlaAlaNHOH systems.

As it is clearly seen in Table 4, identical equilibrium models were determined for the [(η^6^-*p*-cym)Ru(H_2_O)_3_]^2+^–AlaAlaNHOH and [(η^5^-Cp*)Rh(H_2_O)_3_]^2+^–AlaAlaNHOH systems. The only difference is that the stability constants are slightly higher for the complexes with the Ru-containing cation compared to Rh similarly as it was previously found with monohydroxamic acids and with aminohydroxamic acids [13,31]. Surprisingly, as Table 4 shows, apart from [MHL]^2+^, only dinuclear complexes are formed in these systems, which predominate even at 1:1 metal ion to ligand ratio (see inset in Figure 5) and at ligand excess. The suggested coordination mode in the only mononuclear protonated complex is shown as structure IV in Scheme 4. (As an example, the binding modes are shown for the complexes formed with [(η^6^-*p*-cym)Ru(H_2_O)_3_]^2+^ but those are the same in the corresponding [(η^5^-Cp*)Rh(H_2_O)_3_]^2+^ containing complexes.) 

In IV, the hydroxamate chelate (having rather high conditional stability even under strongly acidic conditions with these metal ions [13]), together with a water molecule saturate the three coordination sites of a half-sandwich cation, while the amino group is protonated. The hydroxamate and, after deprotonation of the hydroxamate-NH, the hydroximate chelate is not displaced by other coordinating donors of this dipeptide-based primary hydroxamic acid remaining coordinated in the whole studied pH-range. That is the reason why the amino-N, the peptide moiety, and the hydroximate-N can only coordinate to another metal cation, forming dinuclear complexes in this way. The dominance of the dinuclear species might cause the high complexity of the ^1^H-NMR spectra for the signals of the methyl groups of the Cp* ligand and especially that of the *p*-cymene aromatic protons above pH ca. 4. To illustrate this, pH-dependent chemical shifts of the *p*-cymene aromatic protons are shown in Figure S2. Additional support for the dominant formation of dinuclear complexes with AlaAlaNHOH in these systems was obtained by ESI-MS with the direct identification of [M_2_H_−2_L]^+^ at and above pH = 8.01 (see Figure S3 for the measured and calculated spectra of this species).

The suggested binding modes in the dinuclear complexes [M_2_L]^3+^, [M_2_H_−1_L]^2+^, [M_2_H_−2_L]^+^, and [M_2_H_−3_L] are shown as structures V, VI, VII and VIII, respectively, in Scheme 4.

**Figure 6:**
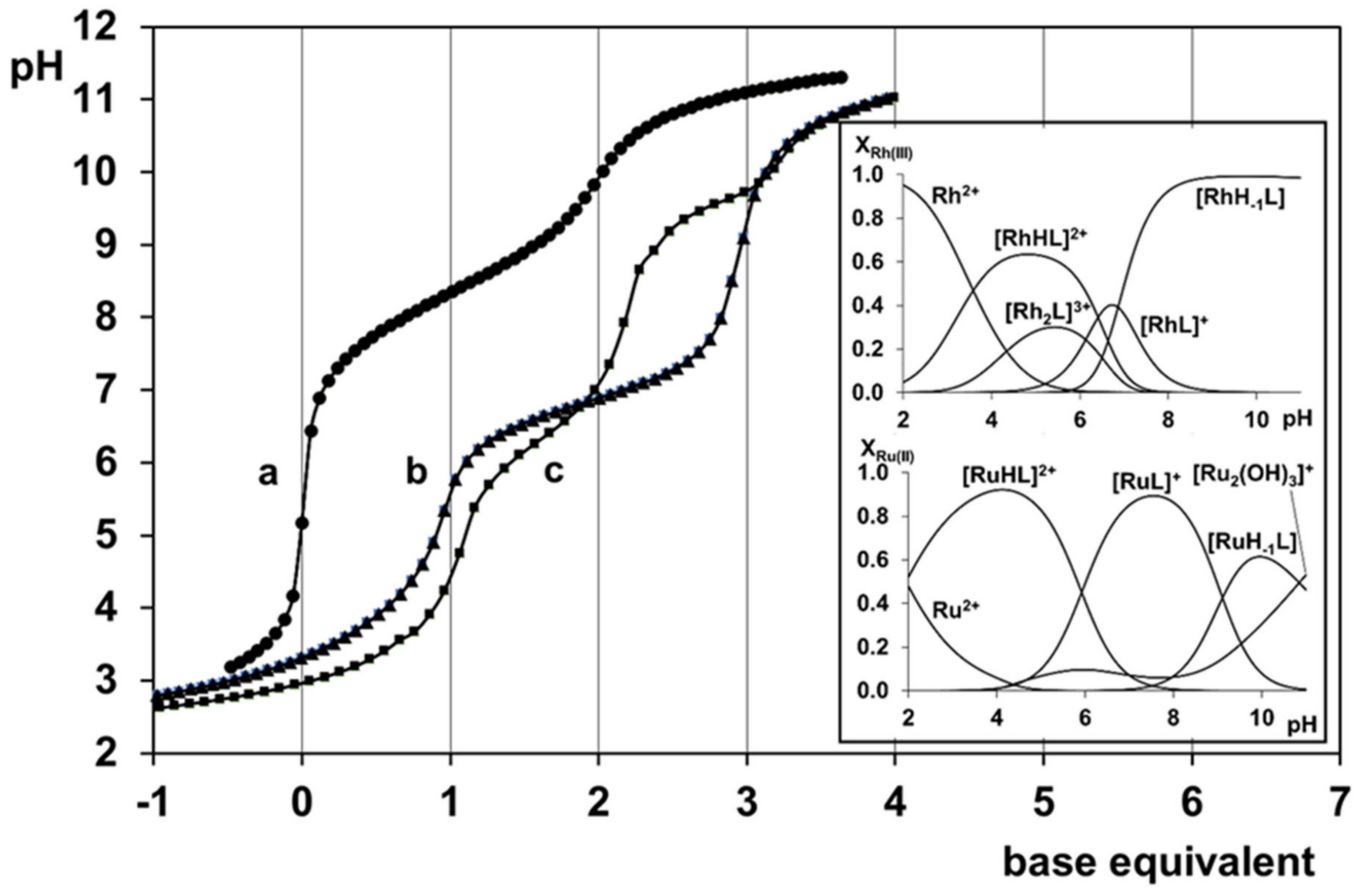
Representative titration curves for the (**a**) H^+^–AlaAlaN(Me)OH, (**b**) [(η^5^-Cp*)Rh(H_2_O)_3_]^2+^–AlaAlaN(Me)OH, and (**c**) [(η^6^-*p*-cym)Ru(H_2_O)_3_]^2+^–AlaAlaN(Me)OH systems with their related concentration distribution curves in inset at 1:1 metal ion to ligand ratio. (c_L_ = 1.80 mM; negative base equivalent refers an excess of acid in the sample; ”Rh” stands for [(η^5^-Cp*)Rh(H_2_O)_3_]^2+^; ”Ru” stands for [(η^6^-*p*-cym)Ru(H_2_O)_3_]^2+^).

Although the complexation processes with the secondary AlaAlaN(Me)OH were somewhat slower compared to those with the primary AlaAlaNHOH, they were still fast enough to perform direct pH-metric titrations. Because for AlaAlaN(Me)OH, the substitution of the hydrogen at the hydroxamic-nitrogen by a methyl moiety hinders the formation of either the hydroximate chelate with very high stability or the (NH_2_,N_amide_,N_hydr_) tridentate coordination mode (both playing a very important role in the interaction between AlaAlaNHOH and the two studied metal ions, as seen in structures VI, VII and VIII), the significant differences between the equilibrium models obtained with the primary and secondary dipeptide hydroxamic acids (Table 4) are understandable. Apart from the minor species, [M_2_L]^3+^, with [(η^5^-Cp*)Rh(H_2_O)_3_]^2+^ in an intermediate pH-range, only mononuclear complexes are seen in Table 4 with the secondary ligand. The model was also supported by the ESI-MS results. The existence of [ML]^+^ and [MH_−1_L] were detected (the latter in its adduct with K^+^) in both systems, while [M_2_L]^3+^ was detected only with [(η^5^-Cp*)Rh(H_2_O)_3_]^2+^. The corresponding m/z values are shown in Table S1. Despite the almost identical equilibrium models, the binding modes cannot be the same in all of the suggested complexes with AlaAlaN(Me)OH and the two half-sandwich cations because the pH-metric titration curves indicate significant differences. This is demonstrated in Figure 6, where the titration curve of the ligand, and those registered at 1:1 metal ion to ligand ratio with the two cations are presented. In inset of Figure 6, the concentration distribution curves calculated using the pH-metric results are also shown. 

**Scheme 5:**
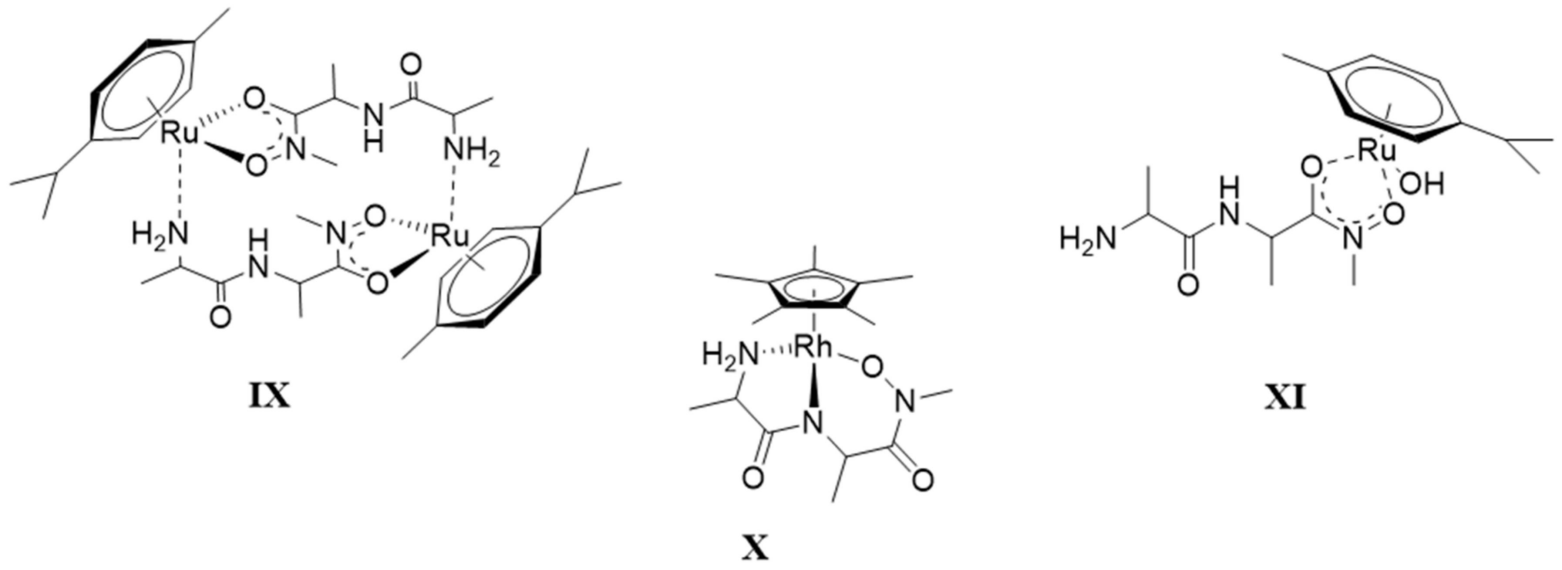
Suggested binding modes for the complexes formed in half-sandwich metal ion–AlaAlaN(Me)OH systems.

As can be seen in Table 4, the stability constants obtained for the [MHL]^2+^ complexes are similar to the values of the corresponding ones with AlaAlaNHOH. This supports the same coordination mode (IV in Scheme 4) in [MHL]^2+^ in all the studied systems. With the secondary ligand, however, this species is formed in a wider pH-range and higher extent than with the primary counterpart (*cf*. the corresponding speciation curves in Insets in Figure 5 and Figure 6). The complex [M_2_L]^3+^, which was found only with the Rh(III)-containing cation, most probably has the binding mode shown in structure V in Scheme 4. Above pH ca. 5 [ML]^+^ is formed in both systems, but predominates only in the [(η^6^-*p*-cym)Ru(H_2_O)_3_]^2+^-AlaAlaN(Me)OH system between pH ca. 6–8.5, while its amount is not significant at all with [(η^5^-Cp*)Rh(H_2_O)_3_]^2+^. Assuming that the terminal-NH_3_^+^ deprotonates in the [MHL]^2+^ ⇌ [ML]^+^ + H^+^ equilibrium process, the p*K* values, that can be calculated using the corresponding stability constants in Table 4 (17.53 − 11.61 = 5.92 for the complex with [(η^6^-*p*-cym)Ru(H_2_O)_3_]^2+^, while 15.89 − 9.48 = 6.41 for the complex with [(η^5^-Cp*)Rh(H_2_O)_3_]^2+^) ^.^are significantly lower than the p*K* of the free ligand (see Table 1). This strongly suggests the coordination of the terminal amino-N in the [ML]^+^ species. However, due to sterical reasons, the simultaneous coordination of the (O,O)_hydr_ chelate and that of the amino-N are not possible at the three available sites of the same metal ion, and the stoichiometry of this species should be [M_x_L_x_]^x+^. The suggested structure for [M_2_L_2_]^2+^ is shown as structure IX in Scheme 5. Unfortunately, the methods applied could not provide direct information for the number of x, additional investigations are necessary to address this problem. 

On the contrary, rather large differences can be expected between the binding modes in [MH_−1_L] formed with the two cations. As the comparison of curves b and c in Figure 6 reveals, the dissociation processes [MHL]^2+^ ⇌ [ML]^+^ + H^+^ and [ML]^+^ ⇌ [MH_−1_L] + H^+^ overlap with each other considerably in the [(η^5^-Cp*)Rh(H_2_O)_3_]^2+^-containing system (the corresponding p*K* values are 6.41 and 6.86 in Table 4), while those are completely separated from each other in the other system (the stepwise p*K* values are 5.92 and 9.11). The reason of this large difference originates from the rather different hydrolytic behaviour of the two half-sandwich cations, being the hydrolysis of the ruthenium-containing cation more pronounced [32]. As a consequence, [MH_−1_L] can be formed with (NH_2_,N_amide_,O_hydr_) tridentate coordination mode in the [(η^5^-Cp*)Rh(H_2_O)_3_]^2+^–AlaAlaN(Me)OH system only (structure X in Scheme 4), while the species with the same stoichiometry is a mixed hydroxido complex for the other metal ion (structure XI in Scheme 5).

### 2.5. Complexation of AlaGlyGlyNHOH or AlaGlyGlyN(Me)OH with the Two Half-Sandwich Type Cations—[(η^6^-p-cym)Ru(H_2_O)_3_]^2+^ or [(η^5^-Cp*)Rh(H_2_O)_3_]^2+^

Unfortunately, conventional pH-metric titrations could only be carried out below pH ca. 6 for the two systems containing one of the studied tripeptide hydroxamic acids and the [(η^6^-*p*-cym)Ru(H_2_O)_3_]^2+^ cation. Above this pH, the processes became too slow to reach pH equilibrium within maximum 30 min (see Materials and Methods section). As a consequence, calculations relating to the stoichiometry and stability constant of the complexes formed in these systems could only be carried out below pH 6. According to the pH-metric titration curves, however, the behaviour of the two ligands toward this half-sandwich cation is significantly different even in this narrow pH-range. For the primary derivative, two equivalents of base are consumed below pH 6 at 1:1 metal ion to ligand ratio, but only one with the secondary counterpart. There is no doubt that one equivalent base, in the pH-range ca. 2.5–4.5, is consumed for the neutralization of the proton released from the hydroxamic function and the (O,O)_hydr_ chelate is formed. Direct proof for this assumption was obtained from the ^1^H-NMR spectra, in which e.g., a new singlet of the C-terminal glycine-CH_2_ protons (designated as “D” protons in the formula of AlaGlyGlyNHOH in Scheme 1) refers to the complex at 3.96 ppm, while the one for the non-complexed ligand is seen at 3.92 ppm. Indication is provided for the coordination of the amino-N in the pH-range of this second base-consuming process by the signal of the “B” protons in the ^1^H-NMR spectra (the new signal belonging to the complex appears at 3.32 ppm). Most probably, due to sterical reasons this process was not observable with AlaGlyGlyN(Me)OH below pH 6 (spectra for this latter system are not presented here). The stoichiometries and the overall stability constants of the complexes yielding the best fit of the titration curves below pH 6 are as follows: [(η^6^-*p*-cym)Ru(H_2_O)_3_]^2+^-AlaGlyGlyNHOH: log*β*_[MHL]2+_ = 17.19(2), log*β*_[ML]+_ = 13.33(3); [(η^6^-*p*-cym)Ru(H_2_O)_3_]^2+^-AlaGlyGlyN(Me)OH: log*β*_[MHL]2+_ = 17.14(2). 

^1^H-NMR measurements were also performed up to pH ca. 11 in these two systems, but detailed evaluation of them was not made because the spectra registered one hour after the preparation of the samples, as was often the case (see Materials and Methods section), which clearly did not refer to the equilibrium state in these samples above pH ca. 6. This was unambiguously supported by control measurements on a few samples where up to five days from the first registration the spectra were monitored again. 

**Figure 7:**
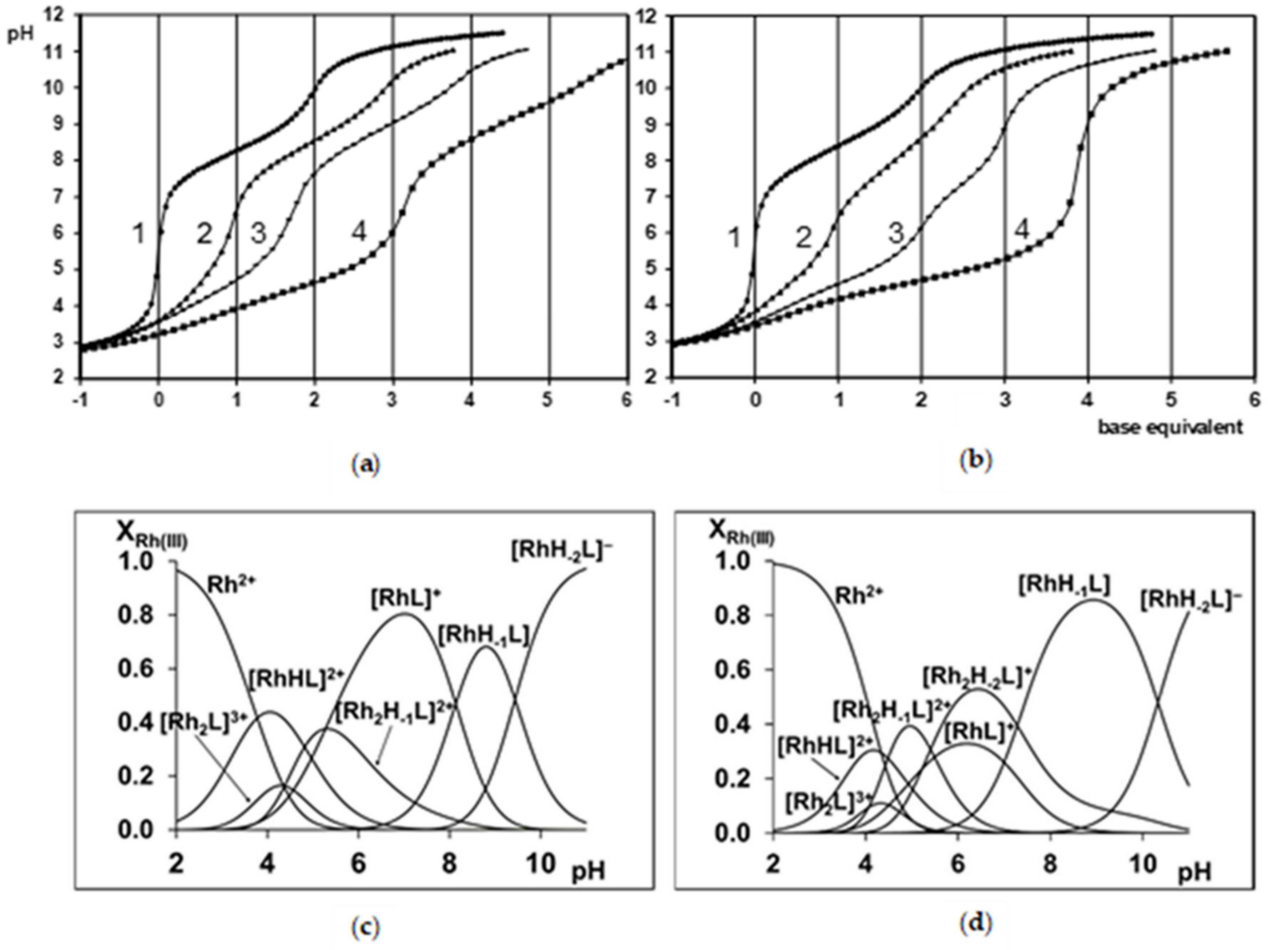
Titration curves with (**a**) AlaGlyGlyN(Me)OH and (**b**) AlaGlyGlyNHOH for (1) the H^+^‒ligand and the [(η^5^-Cp*)Rh(H_2_O)_3_]^2+^‒ligand systems at (2) 1:2, (3) 1:1, and (4) 2:1 ratios (c_L_ = 1.62–1.82 mM, I = 0.20 M KNO_3_); Calculated speciation curves for (**c**) the [(η^5^-Cp*)Rh(H_2_O)_3_]^2+^‒AlaGlyGlyN(Me)OH (c_L_ = 1.82 mM) and (**d**) [(η^5^-Cp*)Rh(H_2_O)_3_]^2+^‒AlaGlyGlyNHOH (c_L_ = 1.62 mM) systems at 1:1 ratio (negative base equivalent refers an excess of acid in the sample, “Rh” stands for [(η^5^-Cp*)Rh(H_2_O)_3_]^2+^).

Fortunately, the processes were fast enough to perform pH-metric titrations in the [(η^5^-Cp*)Rh(H_2_O)_3_]^2+^–AlaGlyGlyN(Me)OH or –AlaGlyGlyNHOH systems, as demonstrated by Figure 7a,7b, respectively. The stoichiometry and overall stability constants of the complexes providing the best fit of the titration curves, together with the suggested binding modes of the complexes, are listed in Table 5. Concentration distribution curves calculated for the complexes formed in the above systems at 1:1 metal ion to ligand ratio using the corresponding stability constants are shown in Figure 7c,d, respectively. 

**Figure 8:**
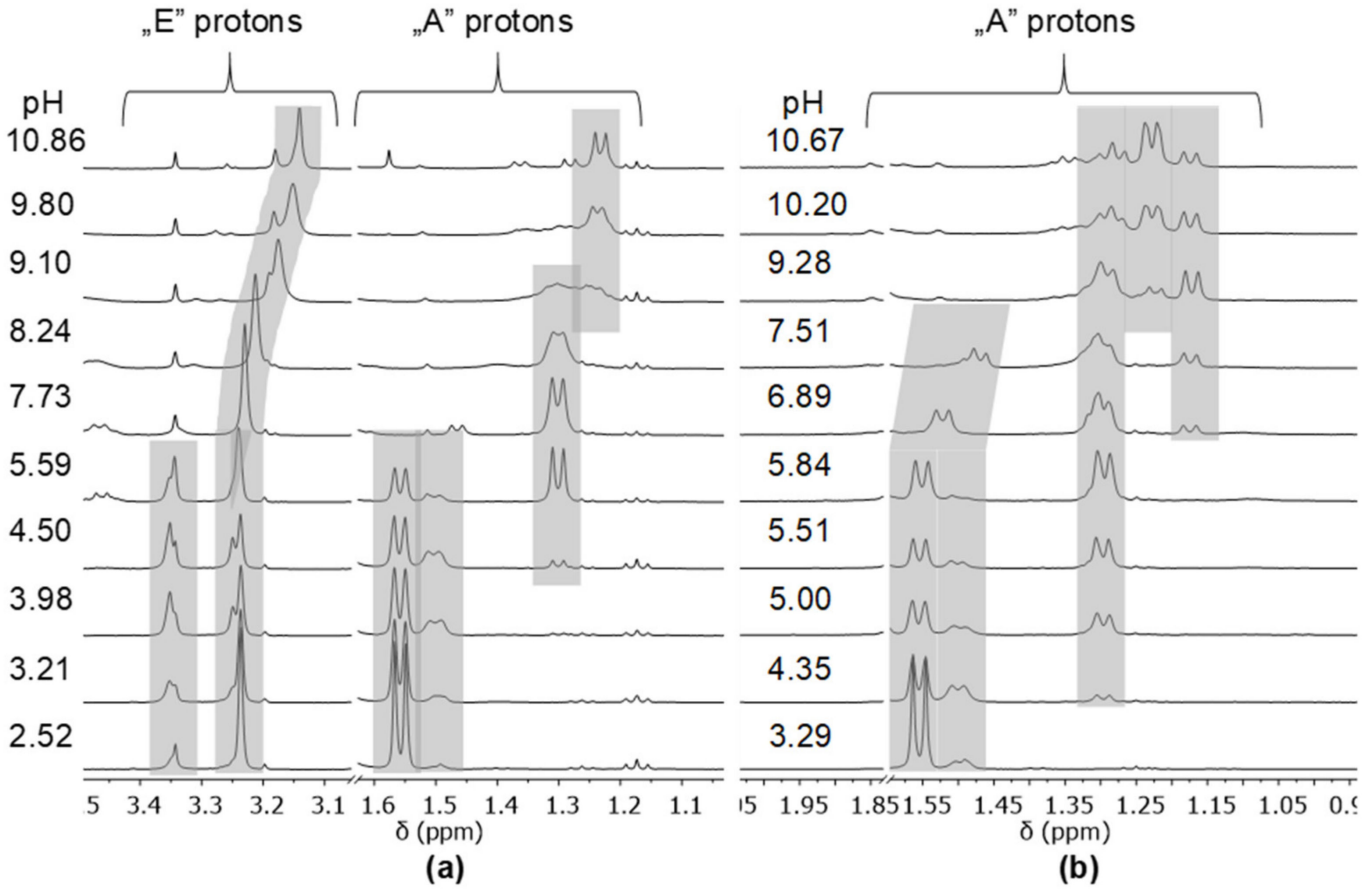
^1^H-NMR signals of E (singlets) and A (doublets) protons of the species formed in the (**a**) [(η^5^-Cp*)Rh(H_2_O)_3_]^2+^–AlaGlyGlyN(Me)OH and (**b**) [(η^5^-Cp*)Rh(H_2_O)_3_]^2+^–AlaGlyGlyNHOH systems at 1:1 metal ion to ligand ratio as a function of pH (c_L_ = 5.00 mM; ^1^H-NMR signal at 3.34 ppm refers to methanol impurity in the ligand).

Although some noticeable differences can be seen between the titration curves registered for the systems containing the primary and secondary ligands, Table 5 shows almost the same equilibrium models for the two systems (except the species [M_2_−__H_2_L]^+^, which was formed only with the primary derivative). A comparison of the two speciation profiles in Figure 7c,d however, indicated significant differences, especially in the intermediate pH-range (ca. 5–8). These differences are also supported by the ^1^H-NMR spectra registered for the systems at 1:1 and 2:1 metal ion to ligand ratios. To illustrate this, Figure 8 shows the pH-dependence of the chemical shifts of the methyl (A) and (E) protons of AlaGlyGlyN(Me)OH as well as (A) protons of AlaGlyGlyNHOH in presence of equimolar amount of [(η^5^-Cp*)Rh(H_2_O)_3_]^2+^ in the pH-range ca. 2–11. Figure 8 reveals that individual signals belong to each of the complexes as well as to the free ligands in these systems. Consequently, the NMR results provided clear support to the speciation model and to the determination of the most likely binding modes in the complexes. (As an example, the registered spectra as a function of pH for the [(η^5^-Cp*)Rh(H_2_O)_3_]^2+^–AlaGlyGlyN(Me)OH system are shown in the Supplementary Figure S4.)

The conclusions, which can be drawn from a comparison of the equilibrium models/stability constants in Table 5, from the concentration distribution curves shown in Figure 7c,d and from the selected ^1^H-NMR signals presented in Figure 8 are as follows: With the secondary derivative, the complexation starts above pH ca. 2 with the formation of [MHL]^2+^, in which (O,O)_hydr_ coordination mode is supported (a singlet in Figure 8a at 3.35 ppm belongs to the N-methyl (E) protons situating in the complexed ligand, while another one at 3.22 ppm to the protonated free ligand. The doublet at 1.55 ppm refers to the methyl protons of Ala (A)). Upon increasing the pH, first, the relative intensity of the singlet at 3.35 ppm increases up to pH ca. 4.5 and then starts to decrease and disappears by pH ca. 7. This suggests the displacement of the hydroxamate from the coordination sphere by pH 7, and, since the non-complexed hydroxamic function exists in its protonated form below and ca. pH 7, protonation of the displaced hydroxamate function occurs (a significant increase is seen in the intensity of the singlet at 3.22 ppm above pH 7). The appearance of a new doublet (1.55 ppm) at pH = 3.21 indicates the formation of a new species (this should be [M_2_L]^2+^), in which the hydroxamate chelate still exists, but the NH_2_-function is also coordinated to another half-sandwich cation, most probably together with the neighbouring carbonyl-O. A new doublet at 1.30 ppm appears at pH ca. 4.5. Its relative intensity shows a sharp increase upon increasing the pH from 4.5 to 5.5, and some additional increase up to pH ca. 7.5 is observable, too. This signal starts to broaden, and its relative intensity decreases above pH ca. 8 but remains in the spectra up to as high as pH 9.8. Consumption of three equivalents of base and existence of three different species are shown in Figure 7a,c respectively, within the pH-range, where the doublet at 1.30 ppm exists. The three species, which are formed within the mentioned pH-range are the [M_2_H_−1_L]^2+^, [ML]^+^, and [MH_–1_L]. As it is evident from the above NMR results, the coordination mode of the amino terminus of the tripeptide derivative in these three complexes is similar to each other and occurs, most probably, via (NH_2_,N_amide_,CO). The differences between the coordination modes of these complexes are due to the C-terminal end, where the hydroxamic function is situated. This latter function is deprotonated and coordinated to a second metal ion in [M_2_H_−1_L]^2+^, it is non-coordinated and protonated in [ML]^+^ (the formula of this species is in fact [MH(H_−1_L)]^+^), while it is non-coordinated and deprotonated in [MH_−1_L]). (The deprotonation of the non-coordinated hydroxamic function is clearly seen in Figure 8a from the upfield shift of the resonance of the N-methyl singlet (E) from 3.22 ppm to 3.10 ppm between pH ca. 7–9.) The thermodynamically most stable complex, [MH_−2_L]^–^, in this system is present above pH 8 with the coordination mode of (NH_2_,N_amide_,N_amide_), via two joined five-membered chelates. 

**Scheme 6:**
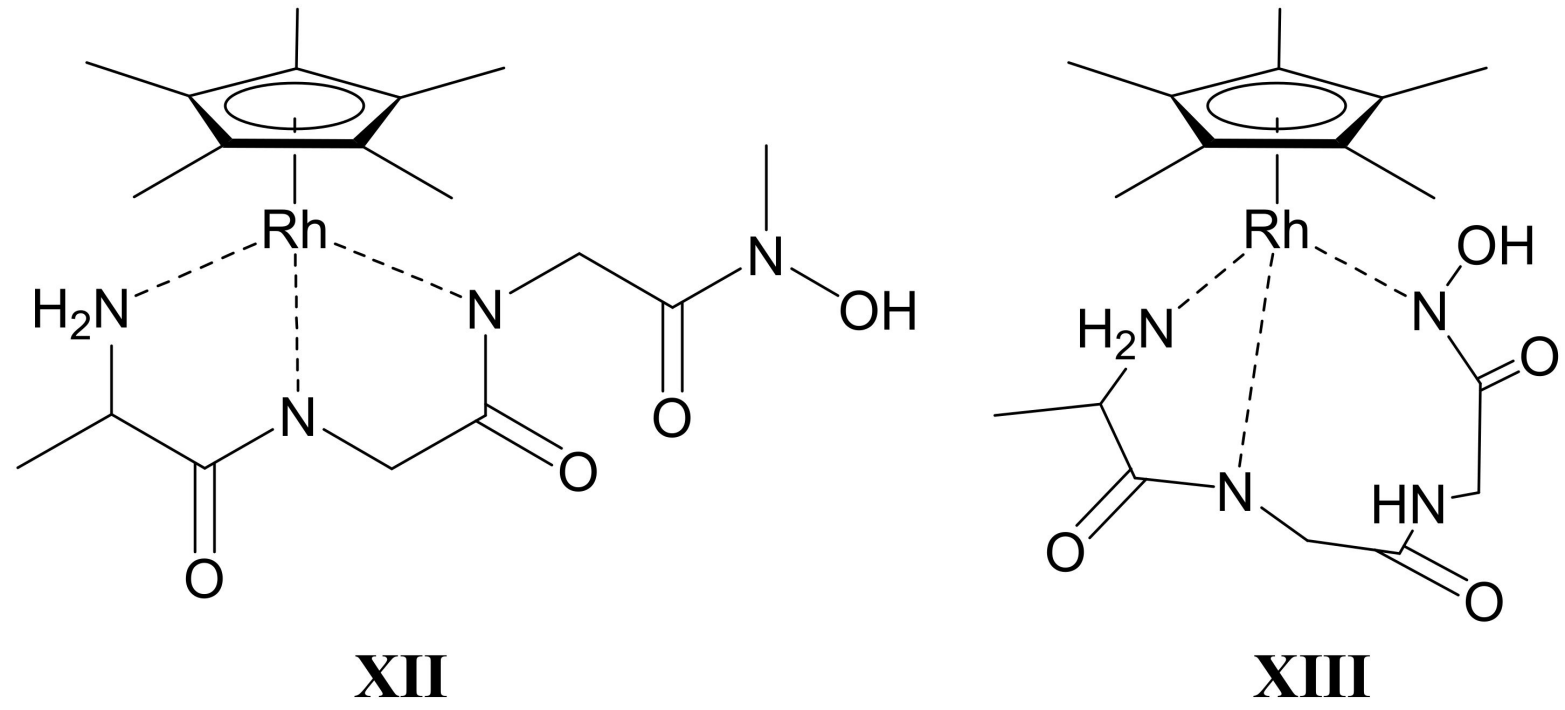
Suggested binding modes for the [MH_−1_L] complexes formed in the [(η^5^-Cp*)Rh(H_2_O)_3_]^2+^–AlaGlyGlyN(Me)OH (structure **XII**) and [(η^5^-Cp*)Rh(H_2_O)_3_]^2+^–AlaGlyGlyNHOH (structure **XIII**) systems.

Compared to the secondary derivative, the primary counterpart AlaGlyGlyNHOH has an additional and strong competitor donor, N_hydr_ in its deprotonated form. Consequently, in this ligand, the number of the N-donors, which can be in chelatable position to each other, is four (NH_2_, two N_amides_, N_hydr_), which is more than enough to saturate the three available coordination sites of [(η^5^-Cp*)Rh(H_2_O)_3_]^2+^. Although this results in higher variation of the binding modes with the primary ligand compared to the secondary one no difference can be seen between their coordination behaviour at the start of the interaction (pH ca. 2–3), where the (O,O)_hydr_ dominates with both ligands and also the thermodynamically most stable complex, [MH_–2_L]^–^, has the same binding mode (NH_2_, N_amide,_N_amide_) in both cases. The formation of [MH_−2_L]^–^ (*cf.* Figure 7c,d), however, is somewhat hindered with the primary derivative (its formation pH is above 9) compared to the secondary one (pH ~ 8). This difference clearly supports the importance of the N_hydr_ coordination in the intermediate pH-range with AlaGlyGlyNHOH. The most important role of this donor is indicated by the NMR spectra (Figure 8b) in the pH-range ca. 7–10.5, where a new doublet at high field (1.18 ppm) exists. The pH-dependence of its intensity is very similar to the concentration profile of [MH_−1_L] (*cf*. Figure 7d and Figure 8b) suggesting that this signal can be designated to the latter complex, in which, most probably, three nitrogens are coordinated to the metal cation (NH_2_,N_amide_,N_hydr_). The differences between the binding modes in [MH_−1_L] formed with AlaGlyGlyN(Me)OH and AlaGlyGlyNHOH are well observable by the comparison of structures XII and XIII in Scheme 6. 

## 3. Discussion

Based on the solution equilibrium results and on the comparison of them, the following conclusions can be drawn regarding the complexes of primary and secondary hydroxamic derivatives of common di- and tripeptides, AlaAlaNHOH, AlaAlaN(Me)OH, AlaGlyGlyNHOH, and AlaGlyGlyN(Me)OH, with selected 3*d* metals, Fe(III), Ni(II), Cu(II), Zn(II), and 4*d* metals, tetraoxido anion of Mo(VI), half-sandwich type cations of Ru(II) and Rh(III), [(η^6^-*p*-cym)Ru(H_2_O)_3_]^2+^, and [(η^5^-Cp*)Rh(H_2_O)_3_]^2+^:

1. Due to its highest conditional stability out of the possible ones under highly acidic conditions, the hydroxamate-type chelate, (O,O)_hydr_ is the anchor with all the studied metal ions, Mo(VI), Fe(III), Cu(II), Ru(II), and Rh(III), with which the interaction starts at pH ca. 3 or below. The situation is somewhat different with Ni(II) and Zn(II), where the interaction starts at pH ca. 4 or somewhat above. In these latter systems, binding isomers can exist because the coordination starts either via the five-membered (O,O)_hydr_ or five-membered (NH_2_,CO) chelate. For Zn(II), these initial interactions were followed by hydrolytic processes resulting in the formation of mixed hydroxido complexes.
2. Exclusive hydroxamate-type coordination could be detected in the whole pH-range with Fe(III) and Mo(VI) (or also hydroximate-type one in the case of Mo(VI) with primary derivatives at higher pH). No role of the amino-N or peptide amide-N(s) was found in the complexation with these two metals. Consequently, there is no any significant difference between their complexation with the studied dipeptide and tripeptide derivatives. However, significant difference was found between the Mo(VI)-binding ability of the primary and secondary derivatives, the former being much more effective Mo(VI)-chelators. This is due to the significantly higher stability of the doubly deprotonated hydroximato chelate (which was detected in the Mo(VI)–primary derivative ligand systems up to pH ca. 10). On the contrary, the mono-deprotonated (O,O)_hydr_ chelate cannot compete with the hydrolysis of the Mo(VI) above the neutral pH.
3. Out of the investigated metals, Ni(II) showed the highest preference towards the N-donors over the hydroxamate oxygens. This allows only a minor role of the (O,O)_hydr_ at the beginning of the interaction. After that, Ni(II) forms with the primary AlaGlyGlyNHOH in slow cooperative processes a planar, 4N-coordinated (NH_2_,N_amide_,N_amide_,N_hydr_) 1:1 complex with very high stability, which exclusively exists above pH ca. 6. With the secondary counterpart, however, an (NH_2_,N_amide_,N_amide_,O_hydr_)-coordinated planar complex is formed, with slightly lower stability and dominates only above pH ca. 7. The hydrolysis of the coordinated metal ion is hindered in both complexes, but, in the latter system, formation of bis-complexes in small extent can be observed in the presence of ligand excess. Nevertheless, even the secondary AlaGlyGlyN(Me)OH is a more effective ligand for Ni(II) (the situation is the same with Cu(II)) than the previously studied dipeptide derivatives [19,20].
4. Although the binding modes of the most stable Cu(II) complexes with AlaGlyGlyNHOH and with the secondary counterpart, the 4N-coordinated (NH_2_,N_amide,_N_amide,_N_hydr_) and (NH_2,_N_amide,_N_amide,_O_hydr_) planar 1:1 complexes, respectively, are the same as those with Ni(II); moreover their stability constants are significantly higher than those of the corresponding Ni(II) complexes (see Table 2), these species become predominant in the Cu(II)-containing systems in the higher pH-range only (above pH 8 and 9, respectively). This happens because the (O,O)_hydr_ can play a crucial role in the interaction with Cu(II), and this is not the case for Ni(II). As a result of the measurable competition between the different types of potential donors even at neutral pH, the processes become very slow especially in the Cu(II)–primary tripeptide derivative system (too slow to allow conventional titrimetry). There is some dominance of the formation of oligonuclear (mostly dimeric or trimeric) complexes and, mainly with the secondary derivative, the formation of bis-hydroxamato intermediate complexes is also considerable.
5. There are only maximum three sites available for coordination for the studied half-sandwich type Ru(II) and Rh(III) cations. There is no doubt that with both cations and all four ligands the (O,O)_hydr_ anchor-chelate can remain in the coordination sphere up to pH ca. 7 but is displaced by nitrogen donors, if there is no possibility for the formation of the hydroximate-(O,O) chelate, with the doubly deprotonated hydroxamic function. If, however, it can be formed, it remains in the coordination sphere, which is the case with the primary dipeptide derivative AlaAlaNHOH, and the N-donors can only coordinate to another metal ion. Consequently, dinuclear complexes predominate from slightly acidic conditions until the end of the investigated pH-range with both cations. In the most stable complex, the donor set (NH_2_,N_amide_,N_hydr_) saturates the coordination sites of one metal, while the (O,O)_hydr_ chelate and either a water, or above pH ca. 9–10, a hydroxide ion binds to another metal ion. On the contrary, mononuclear complexes dominate with the secondary counterpart, AlaAlaN(Me)OH, being the most stable an (NH_2_,N_amide_,O_hydr_)-coordinated complex with the [(η^5^-Cp*)Rh(H_2_O)_3_]^2+^ cation. Due to the significantly higher affinity of [(η^6^-*p*-cym)Ru(H_2_O)_3_]^2+^ toward hydrolysis, it forms a mixed hydroxido complex involving an (NH_2_,N_amide_) chelate and a monodentate OH^−^.
6. The complex formation processes were too slow between [(η^6^-*p*-cym)Ru(H_2_O)_3_]^2+^ and the two tripeptide derivatives, AlaGlyGlyNHOH and AlaGlyGlyN(Me)OH, above pH ca. 6, where the interaction with the N-donors started. Consequently, solution equilibrium measurements were possible only on the [(η^5^-Cp*)Rh(H_2_O)_3_]^2+^-containing systems. With the secondary derivative containing three potential nitrogen donors, the initial (O,O)_hydr_ chelate in the highly acidic pH range is partially replaced by the coordination of the NH_2_ group (most probably together with the neighboring CO) above pH ca. 4. This results in the formation of dinuclear species with mixed (O,O)_hydr_ and (NH_2_, CO) coordination below pH ca. 7–7.5 as minor complexes, but mononuclear species with (NH_2_,N_amide_,N_amide_) binding mode (having the hydroxamate function is uncoordinated) dominate even in this intermediate pH-range.
7. The primary AlaGlyGlyNHOH contains four potential N-donors, which is more than enough to saturate the coordination sphere of a [(η^5^-Cp*)Rh(H_2_O)_3_]^2+^ cation. The results show a measurable competition between the C-terminal N_amide_ and N_hydr_ between pH ca. 7–10, but above this pH, the complex with the (NH_2_,N_amide_,N_amide_) donor set, containing the hydroxamate function in uncoordinated form, has increasing dominance.

## 4. Materials and Methods 

### 4.1. Materials and Stock Solutions

The ligands AlaAlaNHOH, AlaAlaN(Me)OH, AlaGlyGlyNHOH, and AlaGlyGlyN(Me)OH were synthesized as previously described [19,20,21]. The purity and the exact concentration of the ligand stock solutions were determined by pH-potentiometry using the Gran’s method [33].

The metal ion stock solutions were prepared from CuCl_2_·2H_2_O, NiCl_2_·5H_2_O, FeCl_3_·6H_2_O, and Na_2_MoO_4_ (Reanal, Hungary) using doubly deionised and ultra-filtered water obtained from a Milli-Q RG (Millipore, Burlington, MA, USA) water purification system. In the case of Fe(III), the stock solution also contained hydrochloric acid (~0.1 M). ZnO (Reanal, Hungary) was dissolved in diluted HCl to prepare Zn(II) stock solution. The metal ion concentrations were checked gravimetrically via precipitation of the quinolin-8-olate salts, Fe(III) was determined by complexometric titration. The acid content of the Fe(III) stock solution was also estimated using the Gran’s method [33]. 

RuCl_3_·xH_2_O, α-terpinene were purchased from Aldrich (St. Louis, MO, USA), and [(η^5^-Cp*)Rh(µ^2^-Cl)Cl]_2_ was a commercial product from Strem Chemicals (Newburyport, MA, USA). These chemicals were used without further purification. [(η^6^-*p*-cym)Ru(µ^2^-Cl)Cl]_2_ was synthesized and purified according to a literature method [34]. The aqueous solutions of [(η^6^-*p*-cym)Ru(H_2_O)_3_](NO_3_)_2_ and [(η^5^-Cp*)Rh(H_2_O)_3_](NO_3_)_2_ were obtained by removal the chloride ions from the corresponding dimers using stoichiometric amount of AgNO_3_. Metal ion and proton concentrations of these metal ion stock solutions were also checked with the aid of potentiometric titrations.

Carbonate-free KOH solutions of known concentrations (ca. 0.2 M during the pH-metric measurements and ca. 5 M to adjust the pH during the ^17^O-NMR) were used as titrant. HCl and HNO_3_ stock solutions were prepared from their concentrated solutions (both acids and the base were Merck products, Germany), and their concentrations were determined by pH-metric titrations.

### 4.2. Solution Studies

For all of the solution studies, doubly deionised and ultra-filtered water was obtained from a Milli-Q RG (Millipore) water purification system. 

pH potentiometric measurements were carried out at 25.0 ± 0.1 °C, at an ionic strength of 0.20 M KNO_3_ if the metal ion was either [(η^6^-*p*-cym)Ru(H_2_O)_3_]^2+^ or [(η^5^-Cp*)Rh(H_2_O)_3_]^2+^, while in all the other cases, it was at 0.20 M KCl. For the systems containing Ru(II) or Rh(III) and a dipeptide hydroxamic acid as ligand a Mettler Toledo (Switzerland) T50 titrator, if, however, the ligand was a tripeptide hydroxamic acid, a Mettler Toledo DL50 titrator was used for the pH-metric titrations. Both instruments were equipped with a Metrohm (Switzerland) double junction (6.0255.100) combined electrode. In all the other cases a Radiometer pHM 84 instrument equipped with Metrohm combined electrode (type 6.0234.100) and Metrohm 715 Dosimat burette was used for the pH-metry. The electrode systems were calibrated according to Irving et al. [35]. Therefore, the pH-meter readings could be converted into hydrogen ion concentrations. The water ionization constant was p*K*_w_ = 13.75 ± 0.01 under the experimental conditions used. The initial volumes of the samples were 5.00, 10.00, or 15.00 mL, and the ligand concentrations were varied in the range of 1–5 mM and the metal ion to ligand ratios between 2:1–1:5. Typically, three to five ratios were measured in each system. The samples were stirred and completely deoxygenated by bubbling purified argon. Titrations were performed in the pH-range of 2.0–11.0 or until precipitation occurred in equilibrium controlled mode. pH equilibrium was assumed to be reached if a change in the measured potential was less than 0.01 mV within 11 s and 0.1 mV within 90 s for the Mettler Toledo DL50 and Mettler Toledo T50 titrators, respectively. The maximum waiting time for the metal ion–containing systems was 10 min in general, except the systems containing Ru(II) or Rh(III) half-sandwich cations, where it was 30 min due to the quite slow equilibrium processes. If the equilibrium was not reached within these times, the measured value was not used during the calculation. 

In general, the overall stability constants calculated for the complexes relate to the equilibrium

pM + qH + rL ⇌ [M_p_H_q_L_r_] (5)

*β*_pqr_ = [M_p_H_q_L_r_]/[M]^p^[H]^q^[L]^r^ (6)

where “M” stands for Fe^3+^, Ni^2+^, Cu^2+^, Zn^2+^, [(η^6^-*p*-cym)Ru(H_2_O)_3_]^2+^, or [(η^5^-Cp*)Rh(H_2_O)_3_]^2+^ and “L” represents the completely deprotonated forms of the ligands. Because the charges are not always the same, they are omitted in Equation (5). 

In the case of Mo(VI), however, the calculated stability constants refer to Equation (1) given in Section 2.3.

In all calculations, the stability constants were determined via fitting the titration curves by using the PSEQUAD and SUPERQUAD programs [36,37]. In the case of the systems, where measurable competition between the hydrolysis of the metal ions and the complexation processes can occur, the former was taken into account during the calculations by using the following literature data (where the Davies equation was used to take into account the different ionic strengths; negative stoichiometric number of H relates to the metal induced ionization of the coordinated water).

[Fe(OH)]^2+^ log*β*_1,−1,0_ = −3.21; [Fe(OH)_2_]^+^ log*β*_1,−2,0_ = −6.73; [Fe_2_(OH)_2_]^4+^ log*β*_2,−2,0_ = 4.09; [Fe_3_(OH)_4_]^5+^ log*β*_3,−4,0_ = −7.58 [38].

[HMoO_4_]^–^ log*β*_1,1,0_ = 4.03; [H_2_MoO_4_] log*β*_1,2,0_ = 6.70; [H_8_(MoO_4_)_7_]^6−^ log*β*_7,8,0_ = 53.18; [H_9_(MoO_4_)_7_]^5−^ log*β*_7,9,0_ = 58.10; [H_10_(MoO_4_)_7_]^4–^ log*β*_7,10,0_ = 62.11; [H_11_(MoO_4_)_7_]^3−^ log*β*_7,11,0_ = 64.54 [30].

[{(η^6^*-p-*cym)Ru}_2_(µ^2^-OH)_3_]^+^ (log*β*_2,−3,0_ = −9.16) [32]

[{(η^5^-Cp*)Rh}_2_(µ^2^-OH)_2_]^2+^ (log*β*_2,−2,0_ = −8.53); [{(η^5^-Cp*)Rh}_2_(µ^2^-OH)_3_]^+^ (log*β*_2,−3,0_ = −14.26) [39].

For the Ru–AlaAlaNHOH system (where Ru stands for [(η^6^-*p*-cym)Ru(H_2_O)_3_]^2+^), the corresponding hydrochloride salt of the ligand was used, therefore the studied samples also contain one equivalent of chloride ion as a second ligand. 

^1^H-NMR titrations were carried out on a Bruker (Billerica, MA, USA) Avance 400 instrument at 25.0 °C at an ionic strength of 0.20 M KNO_3_ for the systems containing Ru(II) or Rh(III) half-sandwich cations and on a Bruker AM360 FT NMR instrument at 0.20 M KCl for the systems containing Zn(II) or Mo(VI). The chemical shifts are reported in ppm (δ_H_) from TSP as internal standard. Titrations were performed using individual samples equilibrated for 1 hour prior to the measurements in D_2_O (99.8%) at c_L_ = 10.0 mM in the case of dipeptide hydroxamic acid containing systems and c_L_ = 5.0 mM for the tripeptide hydroxamic acid containing ones. The metal ion to ligand ratios were 1:1 and 2:1, the pH of the samples was varied in the range of 2.0–11.5. The pH of the samples was set up with NaOD or DNO_3_ solutions in D_2_O. The pH* values (pH meter readings in D_2_O solution of a pH-meter calibrated in H_2_O according to Irving et al. [35]) were converted into pH values using the following equation: pH = 0.936 × pH* + 0.412. [40].

On all the other systems, the ^1^H-NMR measurements were made on Bruker AM 360 by using D_2_O as solvent. For Mo(VI), DSS was used as standard, metal to ligand ratio was 1:3 in all samples, the analytical concentration of the ligand was 0.015 M.

For the Mo(VI)-containing systems, ^17^O-NMR measurements were performed at 1:3 metal ion to ligand ratio at c_L_ = 0.15 M. The solvent was 90% H_2_O 10% D_2_O enriched for ^17^O. Enrichment of the molybdate oxygen atoms to 3% was done by the addition of H_2_^17^O (37.8 %, ISOTEC Inc., Matheson CO, USA) to the samples. The enrichment included the M=O oxygens, while the oxygens of the ligands were not involved in the ^17^O isotope enrichment, being inert for oxygen exchange. Spectra were registered on a Bruker DRX 500 NMR equipment at 67.8 MHz. Spectral widths of 1200 ppm (81.4 kHz) were used, and data for the FID were accumulated in 8k blocks. A 40^o^ pulse angle and 100 ms relaxation delays were used. The spectra were integrated after baseline correction by using WINNMR 960901 software. Chemical shifts refer to the signal of tap water, δ = 0 ppm.

Frozen solution EPR (Electron Paramagnetic Resonance) spectra in aqueous samples of the copper complexes were recorded on a Bruker EMX X-band spectrometer, operating at ~9.4 GHz, equipped with a HP 53150A microwave frequency counter using ^63^CuSO_4_ stock solution. Metallic copper was purchased from JV Isoflex (Moscow, Russia) containing 99.3% ^63^Cu and 0.7% ^65^Cu and converted into the sulfate. The samples for low-temperature measurements contained a few drops of ethylene glycol to ensure good glass formation. The metal ion to ligand ratios varied in the range 1:1–1:5 and c_Cu(II)_ was 0.005 M; the pH of the samples was varied in the range of 3.4–10.2. Increasing the resolution and avoid the aggregation process 10 % ethylene glycol was added to the aqueous copper(II) complex samples.

An HP 8453 or Perkin Elmer Lambda 25 spectrophotometer was used to record the UV-Vis spectra over the range 250–800 nm for copper(II)-, iron(III)-, and nickel(II)-containing systems. The path length was 1 cm. Measurements for the iron(III)-containing samples were carried out on individual samples in which the 0.20 M KCl was partially or completely replaced by HCl. pH values, varying in the range ca. 0.7–1.6, were calculated from the HCl content. The metal ion concentrations were in the range 0.0004–0.0008 M, and the metal ion to ligand ratios were in the range 1:1–1:5. Spectrophotometric titrations were also performed with samples containing Fe(III), Cu(II) or Ni(II) in 0.0004–0.004 M concentrations at metal to ligand ratios ranging from 1:1 to 1:5. The spectrophotometric results were utilized to calculate the stability constants for the Fe(III) complexes formed below pH 2.0. 

ESI-MS measurements were carried out on a Bruker micrOTOF-Q instrument in positive ion mode. The samples were prepared in water, at c_M_ = 0.3 mM. The metal ion to ligand ratios were 1:1 and 2:1 in all cases, the pH of the samples was set up according to the concentration distribution curves by using HNO_3_ or KOH solutions. The temperature of the drying gas (N_2_) was 180 °C, and the pressure of the nebulizing gas was 0.3 bar. The flow rate was 3 μL/min. The spectra were accumulated and recovered by a digitalizer at a sampling rate of 2 GHz. DataAnalysis (version 3.4) was used for the calculations.

## Figures and Tables

**Table 1:** Stepwise dissociation constants (p*K*) of the ligands at 25.0 °C, I = 0.20 M (KCl or KNO_3_)*

	I = 0.20 M KCl		I = 0.20 M KNO_3_	
Ligand	p*K*_1_	p*K*_2_	p*K*_1_	p*K*_2_
H-AlaAlaNHOH^+^	7.66 ^1^	8.88 ^1^	7.77(1)	8.89(1)
H-AlaAlaN(Me)OH^+^	7.74 ^2^	8.74 ^2^	7.77(1)	8.70(1)
H-AlaGlyGlyNHOH^+^	7.71(1)	8.82(1)	7.74(1)	8.82(1)
H-AlaGlyGlyN(Me)OH^+^	7.66(1)	8.63(1)	7.69(1)	8.57(1)

* 3σ standard deviations are in parentheses; ^1^ taken from [19]; ^2^ taken from [20].

**Table 2:** Overall stability constants (log*β*) for the Fe(III), Ni(II), Cu(II), and Zn(II) complexes formed with the investigated tripeptide hydroxamic acids at 25.0 °C, I = 0.20 M (KCl)*.

	AlaAlaNHOH ^1^	AlaAlaN(Me)OH ^2^	AlaGlyGlyNHOH	AlaGlyGlyN(Me)OH
[FeHL]^3+^	17.62 ^a^	18.08 ^a^	17.74(1) ^a^	17.64(1) ^a^
[FeL]^2+^	15.09	14.67	–	–
[FeH_2_L_2_]^3+^	34.57	33.8	34.02(5)	33.70(7)
	34.00 ^a^	34.71 ^a^	34.00(2) ^a^	33.76(1) ^a^
[FeH_3_L_3_]^3+^	49.40	50.01	48.7(2)	48.97(11)
	48.61 ^a^	49.51 ^a^	48.55(3) ^a^	48.16(2) ^a^
[NiHL]^2+^	12.50	–	12.79(3)	11.6(2)
[NiL]^+^	6.30	6.01	–	5.04(7)
[NiH_−1_L]	−1.10	–	–	−3.3(1)
[NiH_−2_L]^−^	−9.57	–	−7.44(2)	−10.78(3)
[NiHL_2_]^+^	–	16.8	–	17.10(9)
[NiL_2_]	–	9.13	–	–
[CuHL]^2+^	14.70	15.02	14.45(7)	14.66(2)
[Cu_2_L_2_]^2+^	23.20	–	–	–
[CuH_−1_L]	4.43	3.04	–	3.52(2)
[CuH_−2_L]^−^	−5.24	−6.63	−5.38(5)	−5.28(2)
[CuH_2_L_2_]^2+^	–	29.17	–	27.6(2)
[CuHL_2_]^+^	–	22.3	–	22.23(9)
[CuL_2_]	–	14.8	–	14.8(1)
[CuH_−1_L_2_]^−^	–	6.3	–	–
[Cu_3_H_−4_L_2_]	5.89	–	–	–
[Cu_3_H_−5_L_2_]^−^	−3.56	–	–	–
[Cu_3_H_−6_L_2_]^2−^	−14.28	–	–	–
[ZnHL]^2+^	12.35	12.00	12.47(2)	11.92(2)
[ZnL]^+^	5.64	5.11	5.07(6)	4.55(2)
[ZnH_−1_L]	−2.58	−3.23	−3.18(2)	−3.46(3)
[ZnHL_2_]^+^	17.52	17.12	–	–

* 3σ standard deviations are in parentheses; ^a^ Determined from UV-Vis data; ^1^ taken from reference [19]; ^2^ taken from reference [20].

**Table 3:** Overall stability constants (log*β*) for the Mo(VI) complexes formed with the peptide hydroxamic acids at 25.0 °C, I = 0.20 M (KCl)*.

Species	AlaAlaNHOH	AlaAlaN(Me)OH	AlaGlyGlyNHOH	AlaGlyGlyN(Me)OH
[MoO_2_(HL)_2_]^2+^	46.5(1)	47.78(7)	48.57(9)	46.44(2)
[MoO_3_(HL)]	23.89(7)	24.52(5)	24.37(7)	23.92(2)
Fitting parameter (mL)*^a^*	0.00317	0.00596	0.00662	0.00226
Number of fitted data	422	461	195	271

* 3σ standard deviations are in parentheses; *^a^* Fitting parameter is the average difference between the calculated and experimental titration curves expressed in the volume of the titrant.

**Table 4:** Overall stability constants (log*β*) for the [(η^6^-*p*-cym)Ru(H_2_O)_3_]^2+^ and [(η^5^-Cp*)Rh(H_2_O)_3_]^2+^ complexes formed with AlaAlaNHOH and AlaAlaN(Me)OH at 25.0 °C, I = 0.20 M (KNO_3_)*.

Species	AlaAlaNHOH		AlaAlaN(Me)OH	
	[(η^6^-*p*-cym)Ru]^2+^	[(η^5^-Cp*)Rh]^2+^	[(η^6^-*p*-cym)Ru]^2+^	[(η^5^-Cp*)Rh]^2+^
[MHL]^2+^	16.09(2)	15.54(1)	17.53(2)	15.89(1)
[ML]^+^	–	–	11.61(3)	9.48(3)
[MH_−1_L]	–	–	2.50(5)	2.62(2)
[M_2_L]^3+^	14.95(10)	13.57(8)	–	14.16(2)
[M_2_H_−1_L]^2+^	11.16(5)	10.07(1)	–	–
[M_2_H_−2_L]^+^	4.63(6)	3.40(3)	–	–
[M_2_H_−3_L]	–4.79(9)	–5.47(4)	–	–
Fitting parameter (mL)^a^	0.00683	0.00417	0.00707	0.00575
Number of fitted data	302	183	201	244
p*K*_MHL_	–	–	5.92	6.41
p*K*_ML_	–	–	9.11	6.86

*3 σ standard deviations are in parentheses; ^a^ Fitting parameter is the average difference between the calculated and experimental titration curves expressed in the volume of the titrant.

**Table 5:** Overall stability constants (log*β*) for the [(η^5^-Cp*)Rh(H_2_O_3_]^2+^ complexes formed with tripeptide hydroxamic acids, together with their suggested binding modes at 25.0 °C, I = 0.20 M (KNO_3_)*.

Species	AlaGlyGlyN(Me)OH		AlaGlyGlyNHOH	
	log*β*	Coordinated Donor Atoms	log*β*	Coordinated Donor Atoms
[MHL]^2+^	15.53(4)	(O,O)_hydr_	15.35(4)	(O,O)_hydr_
[ML]^+^	10.56(7)	(NH_2_, N_amide_)	10.42(6)	(NH_2_, N_amide_)
[MH_−1_L]	2.42(6)	(NH_2_, N_amide_)	3.42(4)	(NH_2_, N_amide_, N_hydr_)
[MH_−2_L]^‒^	–7.02(7)	(NH_2_, N_amide_, N_amide_)	–6.91(3)	(NH_2_, N_amide_, N_amide_)
[M_2_L]^3+^	14.0(1)	(O,O)_hydr_ + (NH_2_, CO)	13.6(1)	(O,O)_hydr_ + (NH_2_, CO)
[M_2_H_−1_L]^2+^	9.62(5)	(O,O)_hydr_ + (NH_2_, N_amide_)	9.53(3)	(O,O)_hydr_ + (NH_2_, N_amide_)
[M_2_H_−2_L]^+^	‒		4.12(3)	(O,O)_hydr_ + (NH_2_, N_amide_, N_hydr_)
Fitting parameter (mL)^a^	0.0123		0.00599	
Number of fitted data	265		256	

*For the log*β* values, 3σ standard deviations are in parentheses; ^a^ Fitting parameter is the average difference between the calculated and experimental titration curves expressed in the volume of the titrant; coordinated water molecules at the third coordination sites are omitted.

## Supplementary Materials

The following are available online at https://www.mdpi.com/1420-3049/24/21/3941/s1: **Figure S1**. EPR spectra recorded at different pH values for the (**a**) ^63^Cu(II)–AlaGlyGlyNHOH and (**b**) ^63^Cu(II)–AlaGlyGlyN(Me)OH systems at 1:1 and 1:2 metal ion to ligand ratios, respectively (c_Cu(II)_ = 5.00 mM); **Figure S2**. pH dependence on the low-field region of ^1^H-NMR spectra of [(η^6^-*p*-cym)Ru(H_2_O)_3_]^2+^–AlaAlaNHOH system at 1:1 metal ion to ligand ratio (c_L_ = 10.0 mM); **Figure S3**. Measured and calculated ESI-MS spectra of [M_2_H_−2_L]+ formed in [(η^6^-*p*-cym)Ru(H_2_O)_3_]^2+^–AlaAlaNHOH system at pH = 8.01; **Figure S4**. pH dependence on the ^1^H-NMR spectra of (**a**) [(η^5^-Cp*)Rh(H_2_O)_3_]^2+^–AlaGlyGlyN(Me)OH = 2:1 system and (**b**) CH_3_ signals of Cp* ligand at different pH values (region of the methyl protons is shown with reduced intensity for an easier interpretation); **Table S1**. Observed and calculated *m*/*z* values of the species formed in the investigated half-sandwich cation–dipeptide hydroxamic acid systems.
