# Supplementary material for: Trends and Exceptions in the Interaction of Hydroxamic Acid Derivatives of Common Di- and Tripeptides with Some 3d and 4d Metal Ions in Aqueous Solution
Source: Molecules. 2019 Oct 31;24(21):3941. doi: 10.3390/molecules24213941 (PMC6864811; doi:10.3390/molecules24213941)
Supplement: Supplementary file 1 [file molecules-24-03941-s001.pdf]

Trends and Exceptions in the Interaction of Hydroxamic Acid Derivatives of Common Di- and Tripeptides with Some 3d and 4d Metal Ions in Aqueous Solution

András Ozsváth [1], Linda Bíró [1], Eszter Márta Nagy [1], Péter Buglyó [1], Daniele Sanna [2] and Etelka Farkas [1,*]

[1] Department of Inorganic and Analytical Chemistry, University of Debrecen, H-4032 Debrecen, Egyetem tér 1, Hungary, ozsvath.andras@science.unideb.hu (A.O.); linda.biro@science.unideb.hu (L.B.); neszma@hotmail.com (E.M.N.); buglyo@science.unideb.hu (P.B.)

[2] Istituto CNR di Chimica Biomolecolare, Trav. La Crucca 3, I-07040 Sassari, Italy, Daniele.Sanna@cnr.it

* Correspondence: efarkas@science.unideb.hu; Tel.: +36-52-512-900

Supporting information

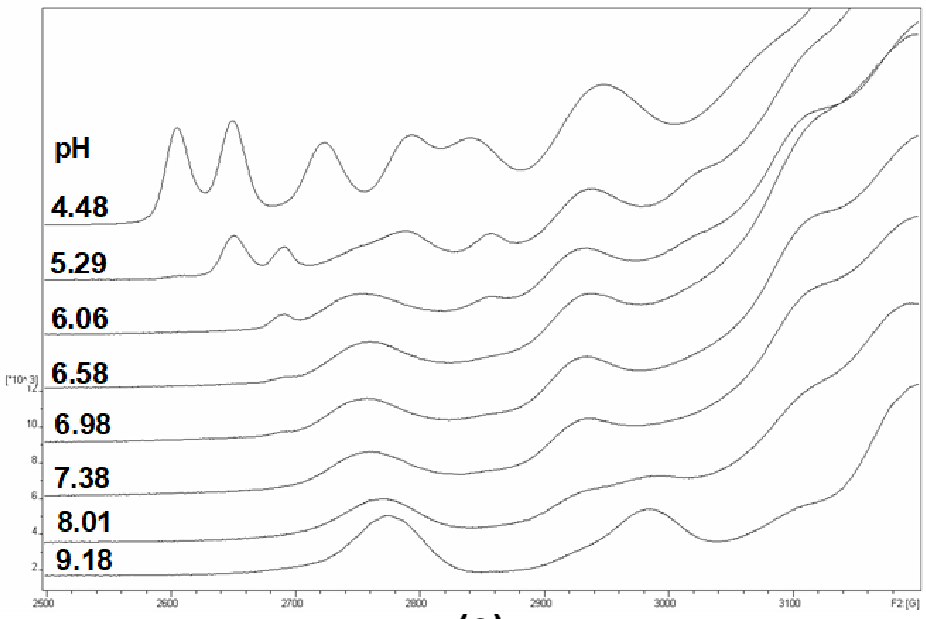

(a)

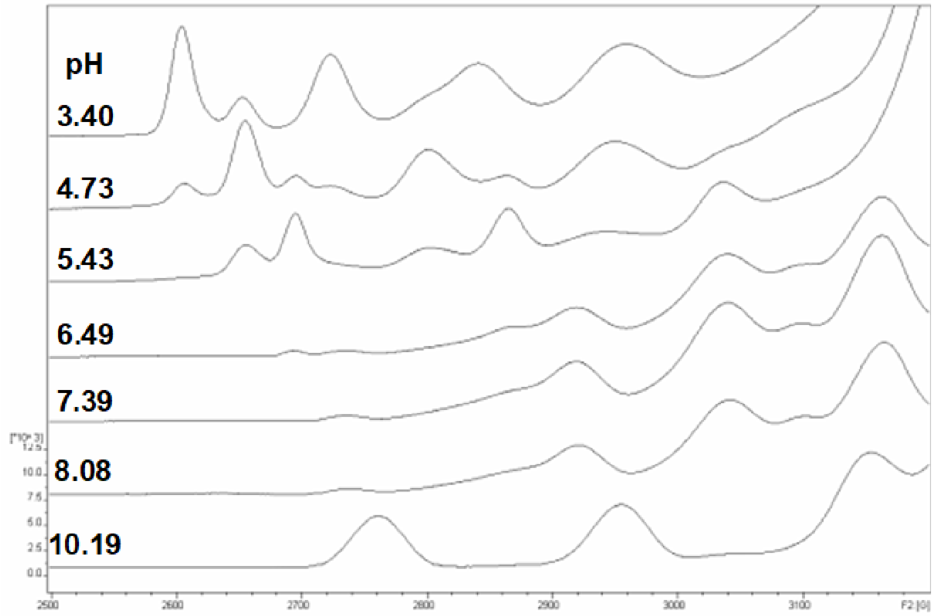

(b)

Figure S1. EPR spectra recorded at different pH values for the (a) ^{63}Cu(II) – AlaGlyGlyNHOH and (b) ^{63}Cu(II) -AlaGlyGlyNMeOH systems at 1:1 and 1:2 metal ion to ligand ratio, respectively ($c_{Cu(II)}$ = 5.00 mM.

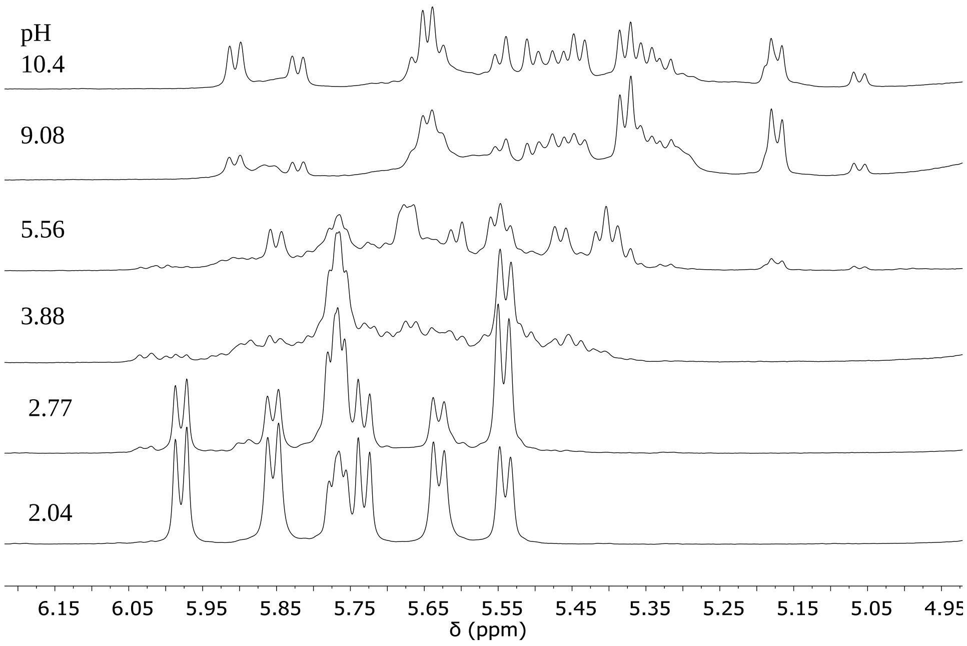

Figure S2. pH dependence on the low-field region of ^{1}H NMR spectra of $[(\eta^6\text{-}p\text{-}cym)Ru(H_2O)_3]^{2+}$ – AlaAlaNHOH system at 1:1 metal ion to ligand ratio (c_L = 10.0 mM)

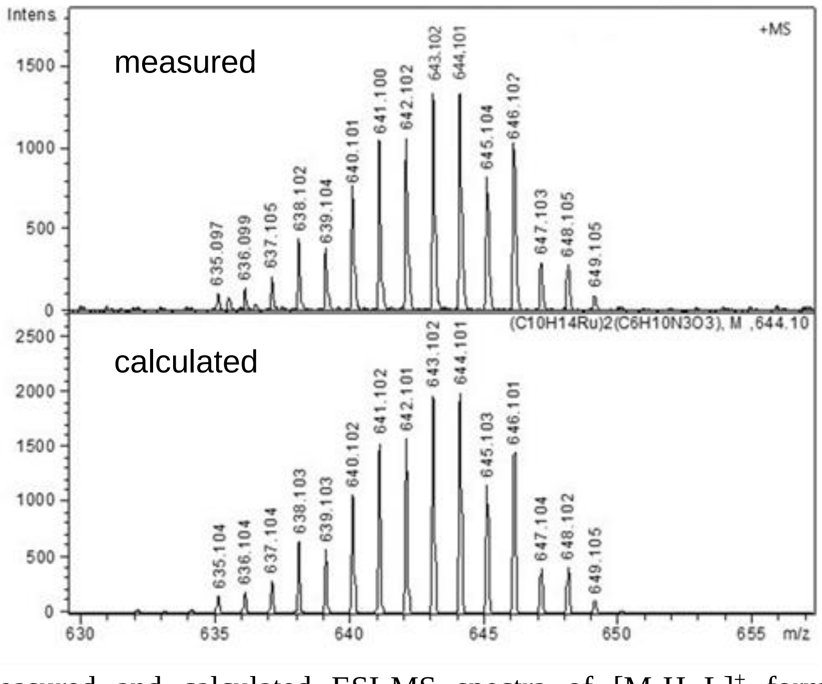

Figure S3. Measured and calculated ESI-MS spectra of $[M_2H_{-2}L]^+$ formed in $[(\eta^6\text{-}p\text{-}cym)Ru(H_2O)_3]^{2+}$ – AlaAlaNHOH system at pH = 8.01.

Table S1. Observed and calculated m/z values of the species formed in the investigated half-sandwich cation - dipeptidehydroxamic acid systems

System	pH	Species	Observed m/z	Calculated m/z
$[(\eta^6\text{-}p\text{-cym})Ru(H_2O)_3]^{2+}$ – AlaAlaNHOH	2.54 – 3.64	$[ML]^+$	410.101	410.102
	8.01	$[MH_{-1}L] + K^+$	448.059	448.057
	8.01	$[M_2H_{-2}L]^+$	643.102	643.102
	8.01 – 10.65	$[M_2H_{-2}L + OH] + K^+$	699.071	699.068
$[(\eta^6\text{-}p\text{-cym})Ru(H_2O)_3]^{2+}$ – AlaAlaN(Me)OH	2.46 – 6.28	$[ML]^+$	424.115	424.117
	6.28 – 10.00	$[MH_{-1}L] + K^+$	462.071	462.073
$[(\eta^5\text{-Cp*})Rh(H_2O)_3]^{2+}$ – AlaAlaN(Me)OH	5.11 – 6.49	$[ML]^+$	426.128	426.126
	6.49 – 8.16	$[MH_{-1}L] + K^+$	464.082	464.082
	5.97	$[M_2LCl_2]^+$	734.084	734.086

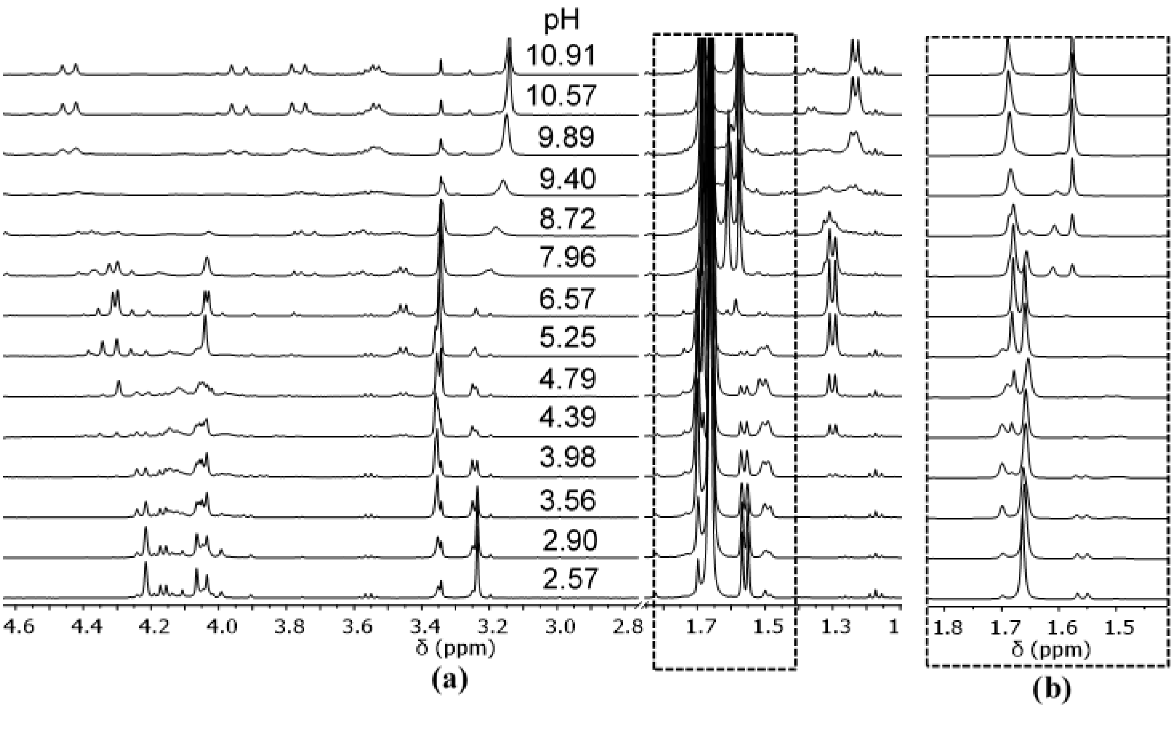

Figure S4. pH dependence on the ^{1}H NMR spectra of (a) $[(\eta^5\text{-Cp*})Rh(H_2O)_3]^{2+}$ – AlaGlyGlyN(Me)OH = 2:1 system and (b) CH_3 signals of Cp* ligand at different pH values (region of the methyl protons is shown with reduced intensity for an easier interpretation)
